# Citalopram exposure of hESCs during neuronal differentiation identifies dysregulated genes involved in neurodevelopment and depression

**DOI:** 10.3389/fcell.2024.1428538

**Published:** 2024-07-11

**Authors:** Mari Spildrejorde, Magnus Leithaug, Athina Samara, Hans Christian D. Aass, Ankush Sharma, Ganesh Acharya, Hedvig Nordeng, Kristina Gervin, Robert Lyle

**Affiliations:** ^1^ PharmaTox Strategic Research Initiative, Faculty of Mathematics and Natural Sciences, University of Oslo, Oslo, Norway; ^2^ Department of Medical Genetics, Oslo University Hospital and University of Oslo, Oslo, Norway; ^3^ Institute of Clinical Medicine, Faculty of Medicine, University of Oslo, Oslo, Norway; ^4^ Division of Clinical Neuroscience, Department of Research and Innovation, Oslo University Hospital, Oslo, Norway; ^5^ Division of Clinical Paediatrics, Department of Women’s and Children’s Health, Karolinska Institutet, Solna, Sweden; ^6^ Astrid Lindgren Children′s Hospital, Karolinska University Hospital, Stockholm, Sweden; ^7^ Department of Biomaterials, FUTURE Center for Functional Tissue Reconstruction, University of Oslo, Oslo, Norway; ^8^ The Flow Cytometry Core Facility, Department of Medical Biochemistry, Oslo University Hospital, Ullevål, Oslo, Norway; ^9^ Department of Cancer Immunology, Institute for Cancer Research, Oslo University Hospital, Oslo, Norway; ^10^ KG Jebsen Centre for B-cell Malignancies, Institute of Clinical Medicine, University of Oslo, Oslo, Norway; ^11^ Precision Immunotherapy Alliance, University of Oslo, Oslo, Norway; ^12^ Division of Obstetrics and Gynecology, Department of Clinical Science, Intervention and Technology (CLINTEC), Karolinska Institutet, Solna, Sweden; ^13^ Center for Fetal Medicine, Karolinska University Hospital, Solna, Sweden; ^14^ Pharmacoepidemiology and Drug Safety Research Group, Department of Pharmacy, University of Oslo, Oslo, Norway; ^15^ Centre for Fertility and Health, Norwegian Institute of Public Health, Oslo, Norway

**Keywords:** citalopram, depression, epigenetics, neurodevelopment, human embryonic stem cells, single-cell RNA-seq, DNA methylation, multi-omics

## Abstract

Selective serotonin reuptake inhibitors (SSRIs), including citalopram, are widely used antidepressants during pregnancy. However, the effects of prenatal exposure to citalopram on neurodevelopment remain poorly understood. We aimed to investigate the impact of citalopram exposure on early neuronal differentiation of human embryonic stem cells using a multi-omics approach. Citalopram induced time- and dose-dependent effects on gene expression and DNA methylation of genes involved in neurodevelopmental processes or linked to depression, such as *BDNF*, *GDF11*, *CCL2*, *STC1*, *DDIT4* and *GAD2*. Single-cell RNA-sequencing analysis revealed distinct clusters of stem cells, neuronal progenitors and neuroblasts, where exposure to citalopram subtly influenced progenitor subtypes. Pseudotemporal analysis showed enhanced neuronal differentiation. Our findings suggest that citalopram exposure during early neuronal differentiation influences gene expression patterns associated with neurodevelopment and depression, providing insights into its potential neurodevelopmental impact and highlighting the importance of further research to understand the long-term consequences of prenatal SSRI exposure.

## 1 Introduction

Depression and anxiety disorders have been associated with impaired serotonergic neurotransmission (Pollock, 2001). In the adult brain, serotonin regulates stress responses, cognition, attention, emotion, nociception, sleep and arousal (Brummelte et al., 2017). During fetal neurodevelopment, serotonin acts as a trophic factor and plays a crucial role in regulation of cell growth, differentiation, migration, myelination and synaptogenesis (Brummelte et al., 2017). Selective serotonin reuptake inhibitors (SSRIs) comprise a class of antidepressants that impede the reuptake of serotonin from the synaptic cleft of the pre-synaptic cell, thus restoring extracellular serotonin levels and increasing serotonergic neurotransmission (Sangkuhl et al., 2011). Consequently, there is the possibility that SSRI-exposure during early embryonic development could affect important neurodevelopmental pathways associated with serotonin signalling.

SSRIs are the first-choice class of antidepressants during pregnancy in many countries, and the reported use during pregnancy ranges from 1%–7% in European countries and 5%–8% in North America (Cooper et al., 2007; Mitchell et al., 2011; Jimenez-Solem et al., 2013; Charlton et al., 2015; Zoega et al., 2015). In Nordic countries, citalopram and its enantiomer escitalopram are among the most prescribed SSRIs to pregnant women (Nordeng et al., 2012; Charlton et al., 2015; Zoega et al., 2015). Some evidence of altered behaviour in offspring has been reported in rodent studies of early SSRI exposure (Ramsteijn and Olivier, 2020), whereas the effect on human development and, in particular, long-term neurodevelopmental outcomes is conflicting (Hjorth et al., 2019). While some epidemiological studies have indicated increased risk of depressive symptoms, social-behavioural disturbances (Klinger et al., 2011; Hanley et al., 2015; Malm et al., 2016; Lupattelli et al., 2018), Attention Deficit/Hyperactivity Disorder (ADHD) and Autism Spectrum Disorder (ASD) (Kobayashi et al., 2016; Andalib et al., 2017; Jiang et al., 2018; Morales et al., 2018), others have not (Jiang et al., 2018; Morales et al., 2018). Moreover, the methods and materials, as well as sample size and quality of these studies varies profoundly.

Environmental exposures during pregnancy may disrupt normal neurodevelopment and modulate the risk of neurodevelopmental disorders (NDDs) in the child. Epigenetic modifications, including DNA methylation (DNAm), have been proposed as mechanisms for this link (Kundakovic and Jaric, 2017). Epigenetic modifications are essential for cellular differentiation and fetal neurodevelopment. Prenatal exposures may impact these epigenetic modifications, inducing long-term adverse effects on brain structure and function (Kundakovic and Jaric, 2017). However, studies assessing the causal underlying mechanisms involved in prenatal exposure to SSRIs and increased risk of altered neurodevelopment are sparse. Recently, we identified epigenetic patterns (i.e., DNAm) in cord blood at birth from children exposed to maternal depression and (es)citalopram during pregnancy and neurodevelopmental trajectories in early childhood (Olstad et al., 2023).

SSRIs have previously been studied at therapeutic concentrations using *in vitro* neuronal differentiation models. In differentiating human hippocampal progenitors, exposure to sertraline for 10 days increased neuronal differentiation (Anacker et al., 2011). In human cortical spheroids, chronic exposure to fluoxetine reversibly altered neuronal activity but did not induce changes to synapse formation (Tate et al., 2021). Further, in human iPSC-derived brain organoids, exposure to paroxetine for 8 weeks induced changes to neurite outgrowth, synaptic markers, myelination and cell composition (Zhong et al., 2020). However, to the best of our knowledge, there are no studies that investigate the effect of long-term exposure of citalopram to *in vitro* human neuronal differentiation.

In the present study, we aimed to identify the effect of citalopram exposure during early neurodevelopment using a recently published *in vitro* platform of neuronal differentiation of human embryonic stem cells (hESCs) towards telencephalic neurons, corresponding to first trimester human development (Samara et al., 2022a; 2022b). This model for studying the effects of maternal medication intake on early fetal brain development provides a unique opportunity to study the effect on gene expression and DNAm in otherwise inaccessible window of development. We focused specifically on citalopram exposure during early pregnancy as this is considered a susceptible period for neurotoxicity, i.e., the foundation of neurodevelopment is laid in the first trimester (Moore et al., 2016). This is the time period with highest prevalence of antidepressant use in pregnancy (Nordeng et al., 2012; Zoega et al., 2015), when many women could be unaware of their pregnancy status. A multi-omics approach with single-cell RNA sequencing (scRNAseq), bulk RNAseq and DNAm was used to investigate citalopram time- and dose-specific effects on gene expression and DNAm during early neuronal differentiation, assessing the potential molecular neurodevelopmental effects of citalopram.

## 2 Materials and methods

Critical reagents and resources are displayed in Supplementary Table S1. All original code can be found at https://github.com/maspil/Citalopram_multiomics.

### 2.1 hESC maintenance

Human embryonic stem cells HS360 (Karolinska Institutet, Sweden, RRID:CVCL_C202) (Ström et al., 2010; Main et al., 2020) were cultured as previously described (Samara et al., 2022a). Briefly, cells were maintained in Essential eight medium (E8, ThermoFisher) on Geltrex (ThermoFisher) pre-coated 6-well culture plates.

### 2.2 Neuronal differentiation of hESCs and exposure to citalopram

To study the *in vitro* effects of citalopram, HS360 hESCs cells were differentiated according to Samara and Spildrejorde et al., 2023; Samara et al., 2022a) with minor changes. Briefly, 70,000 cells/well were seeded in Geltrex pre-coated 12-well plates at Day 0. During Days 1–13, media was changed daily: cells were exposed to media only (control) or media containing citalopram (Sigma-Aldrich). The therapeutic concentration range of citalopram in plasma is considered to be 150–340 nM (Hiemke et al., 2018), however lower concentrations have been measured in cord blood (Hendrick et al., 2003; Rampono et al., 2009; Paulzen et al., 2017). Thus, to cover concentrations relevant for the *in vivo* situation, cells were exposed to 50, 100, 200 or 400 nM citalopram. Neural induction started at Day 1 using base medium (Advanced DMEM/F12 (ThermoFisher), 1% GlutaMAX (GIBCO), 1% Penicillin/Streptomycin (ThermoFisher), 1% N2 supplement (ThermoFisher)) containing 10 µM SB431542 (Sigma-Aldrich), 100 nM LDN-193189 (STEMCELL Technologies) and 2 µM XAV939 (STEMCELL Technologies). At Day 7, cells were seeded at 525,000 cells/well on 12-well culture plates sequentially coated with polyornithine (Sigma-Aldrich), fibronectin (ThermoFisher) and Geltrex, using base medium containing 1% B27 supplement through till Day 13. Cells were harvested for downstream analysis at Day 0, 6, 10 and 13.

### 2.3 Cell viability assay

HS360 cells were washed twice with PBS and collected using Accutase (STEMCELL technologies) and seeded at 20,000 cells/well in Geltrex-coated 96-well plates and incubated in E8 containing 10 µM Rock inhibitor (Y-27632, STEMCELL technologies) at 37°C/5% CO_2_ for 24 h. Media was then changed to E8 alone (control) or E8 containing 0.025, 0.05, 0.1, 0.2, 0.4, 0.8, 3.2, 12.8, 51.2 or 204.8 μ M citalopram (Sigma-Aldrich) in quintuplicate wells. Cells were incubated for 24 h at 37°C/5% CO_2_ and cell viability was assessed using CellTiter-Glo^®^ Luminescent Cell Viability Assay (Promega) according to manufacturer´s instructions.

### 2.4 Flow cytometry

HS360 cells (Day 0) and cells differentiated to Day 13 were washed twice with PBS, collected using Accutase and resuspended in wash medium (Day 0: E8, Day 13: Ad. DMEM). Cells were then centrifuged (300 x *g* for 4 min), supernatants were removed and washed in PBS (300 x *g* for 4 min). Cells used with intracellular markers against SOX1, β3-Tubulin and OCT4, were fixed as follows: Supernatants were removed, cells were resuspended in 1 mL/1 × 10^7^ cells Cytofix Fixation Buffer (BD Biosciences) and incubated for 20 min at room temperature (RT) in the dark. To permeabilize the cell membranes, cells were washed twice (300 x *g* for 4 min) with 1 mL/1 × 10^7^ cells 1X Perm/Wash buffer (BD Biosciences). Cells were then resuspended in 1X Perm/Wash buffer and incubated for 10 min at RT. To block and stain cells, a total volume of 100 µL of cells, antibodies and 1X Perm/Wash buffer were incubated for 30 min in the dark according to concentrations found in Supplementary Table S2. Following incubation, cells were washed twice (300 x *g* for 4 min) in 1 mL 1X Perm/Wash buffer and resuspended in Stain Buffer (FBS) (BD Biosciences) to a concentration of 1-3 x 10^6^ cells/mL and data was acquired by flow cytometry using Accuri C6 (Becton Dickinson, San Jose, CA, United States). Raw data was analysed using FlowJo software v.10. Delta median fluorescence intensity ( ∆ MFI) of gated populations of live/singlets cells was calculated by subtracting the MFI of corresponding isotype control to the MFI of antibody of interest (n = 3). Results were visualized using R software (R Core Team, 2021) and the Tidyverse R package v.1.3.1 (Wickham et al., 2019).

### 2.5 DNA/RNA purification

Cells were washed with PBS and collected by direct lysis in the cell culture well. Genomic DNA and total RNA were isolated from the same biological sample using RNA/DNA purification kit (Norgen Biotek Corp.) and RNA was purified using on-column RNase-Free DNase I Kit (Norgen Biotek Corp.). Nucleic acid quantification was performed using Qubit (ThermoFisher Scientific), purity was measured using Nanodrop 2000 (ThermoFisher Scientific), while RNA and DNA integrity was assessed using 2,100 Bioanalyzer (Agilent Technologies) and 4,200 TapeStation (Agilent Technologies), respectively.

### 2.6 Bulk transcriptome sequencing

The sequencing libraries were prepared with TruSeq Stranded mRNA Library Prep (Illumina, San Diego, CA) according to manufacturer’s instructions. The 89 libraries (Supplementary Table S3) were pooled at equimolar concentrations and sequenced on a NovaSeq 6000 S4 flow cell with 150 bp paired end reads (Illumina, San Diego, CA). The quality of sequencing reads was assessed using BBMap v.34.56 (Bushnell, 2014), and adapter sequences and low-quality reads were removed. The sequencing reads were then mapped to the GRCh38 index using HISAT2 v.2.1.0 (Kim et al., 2015). Mapped paired end reads were counted to features using featureCounts v.1.4.6 (Liao et al., 2014). Differential expression (DE) analysis was conducted in R (R Core Team, 2021) using glmQLFTest function in edgeR v.3.34.1 (Robinson et al., 2010; Zhou et al., 2014; Lun et al., 2016). Benjamini–Hochberg was used to correct for multiple testing and genes were considered significantly differentially expressed with an FDR <0.01 and <0.05 for control (Supplementary Figures S2, S3) and citalopram comparisons, respectively. The linear time-response analysis was conducted using the following model: ∼0+Day * Concentration , where Day was classified as numeric and Concentration was classified as factors. The non-linear time-response analysis was conducted using the following model: ∼0 +Concentration*X, where X was the B-spline basis matrix for a natural cubic spline using Day as predictor variable and degrees of freedom = 2. The linear dose-response analysis was conducted using the following model: ∼0+Day * Concentration , where Day was classified as factors and Concentration was classified as numeric. The non-linear dose-response analysis was conducted using the following model: ∼0+ Day*X, where X was the B-spline basis matrix for a natural cubic spline using Concentration as predictor variable and degrees of freedom = 3. Normalized counts were visualized using the Tidyverse package v.1.3.1 (Wickham et al., 2019). The heatmaps were generated using Pheatmap package v.1.0.12 (Kolde, 2019). The gene set enrichment analysis (GSEA) of pre-ranked gene lists, based on *p*-values and direction of expression change, were performed using GSEA software (Subramanian et al., 2005) identifying biological processes (BP) terms. The size of the analysed gene sets was restricted to 20–1,000 genes, and the chip annotation used was “Human_ENSEMBL_Gene_ID_MSigDB.v7.4. chip”.

### 2.7 DNA methylation analysis

DNAm status of 89 samples (Supplementary Table S3) were assessed using the Infinium MethylationEPIC BeadChip v.1.0_B3 (Illumina, San Diego, CA). Quality control and pre-processing of the raw data was performed in R (R Core Team, 2021) using Minfi v.1.38.0 (Aryee et al., 2014). No samples were removed due to poor quality (detection *p*-values >0.05). Background correction was performed using NOOB method (Triche et al., 2013) and β values (ratio of methylated signal divided by the sum of the methylated and unmethylated signal) were normalized using functional normalization (Fortin et al., 2014). Probes with unreliable measurements (detection *p*-values >0.01) (n = 11,740) and cross-reactive probes (Chen et al., 2013) (n = 43,256) were then removed, resulting in a final data set consisting of 811,233 probes and 89 samples. Probes were annotated with Illumina Human Methylation EPIC annotation 1.0 B5 (hg38). Differential methylation (DM) analysis was performed on the M values (log2 of the β values) using the limma package v.3.48.3 (Ritchie et al., 2015). The linear time-response analysis was conducted using the following model: ∼0+Day ∗ Concentration, where Day was classified as numeric and Concentration was classified as factors. The non-linear time-response analysis was conducted using the following model: ∼0 + Concentration*X, where X was the B-spline basis matrix for a natural cubic spline using Day as predictor variable and degrees of freedom = 2. The dose-response analysis was conducted using the following model: ∼0+Day ∗ Concentration , where Day was classified as factors and Concentration was classified as numeric. The non-linear dose-response analysis was conducted using the following model: ∼0 + Day*X, where X was the B-spline basis matrix for a natural cubic spline using Concentration as predictor variable and degrees of freedom = 3. Benjamini–Hochberg was used to correct for multiple testing using and CpGs were considered significantly differentially methylated with an FDR <0.01 and <0.05 for control (Supplementary Figures S2, S3) and citalopram comparisons, respectively. Gene ontology analysis was performed using top 10,000 DMCs as input to gometh in the missMethyl package v.1.26.0 (Phipson et al., 2016) looking at BP terms.

### 2.8 Collection of cells and single-cell RNA-sequencing

HS360 cells (Day 0) and cells at Day 6, 10 and 13 were washed twice in wells with PBS and detached using Accutase at 37°C for 7–10 min. Cells were then pipetted 10–15 times to separate into single cells and transferred to centrifuge tubes containing the appropriate base media (Day 0: E8, Day 6–13: Ad. DMEM) with 0.04% BSA (Sigma-Aldrich). Cell suspensions were counted and centrifuged at 300 × *g* for 5 min and the supernatant was discarded. Cell pellets were then resuspended in base media containing 0.04% BSA and cell aggregates were filtered out using MACS SmartStrainers (Miltenyi). The cells were counted again and processed within 1 h on the 10x Chromium controller (10x Genomics) according to 10x Genomics protocol CG000315 (Rev A). Approximately 4,200 cells were loaded per channel on the Chromium Next GEM Chip G (10x Genomics) to give an estimated recovery of 2,500 cells. The Chromium Next GEM Single Cell 3ʹ Kit v3.1 (10x Genomics) and Dual Index Kit TT Set A (10x Genomics) were used to generate single-cell (sc)RNA-seq libraries according to the manufacturer’s instructions. Libraries from 16 samples were pooled together at equimolar concentrations and sequenced on a NovaSeq 6000 S1 flow cell (Illumina) with 28 cycles for read 1, 10 cycles for the I7 index, 10 cycles for the I5 index and 90 cycles for read 2.

### 2.9 scRNAseq analysis

The Cell Ranger 4.0.0 Gene Expression pipeline (10x Genomics) was used to demultiplex the raw base-call files and convert them into FASTQ files. The FASTQ files were aligned to the GRCh38 human reference genome (2020-A), and the Cell Ranger count quantified single-cell gene expression using default parameters. Cell Ranger´s estimated number of recovered cells/sample were from 1,113–2,528, with mean reads/cell spanning from 27,000–119 000. Downstream analysis was performed using the R software (R Core Team, 2021). Duplicates, dead cells and cells with greater than five median absolute deviations (MADs) for mitochondrial reads were filtered out using the scater R package (McCarthy et al., 2017) resulting in a total of 20,217 cells (Supplementary Table S3) for downstream analysis. SCTransform with regression of cell cycle genes and mitochondrial content (Tirosh et al., 2016; Hafemeister and Satija, 2019) was used to normalise data. A resolution of 0.55 was used to cluster cells, obtained by determining the optimum number of clusters (cell grouped together sharing similar expression profiles) in the dataset using the Clustree R package (Zappia and Oshlack, 2018). Principal component analysis was performed using the RunPCA function, followed by FindClusters and RunUMAP functions of Seurat package (Stuart et al., 2019) to perform SNN-based UMAP clustering. The SingleR R package (Aran et al., 2019) was used to annotate the cells against a merged reference dataset derived from 1: from a Human Brain dataset (LaManno et al., 2016) and 2: a forebrain organoid dataset (Bhaduri et al., 2020), from the scRNAseq R package. Cell types with <10 cells annotated are excluded from the plots (Figures 5D, E). Slingshot R package was used to create the pseudotime differentiation trajectory (Street et al., 2018). Temporally expressed genes were identified by fitting generalized additive model for each gene using the gam R package (Hastie, 2022) and visualized using pheatmap (Kolde, 2019) and scater (McCarthy et al., 2017) R packages. Differential pseudotime analysis were calculated by removing Day 0 from dataset and looking at differences in pseudotime values between citalopram-exposed cells and control cells using the following methods: compare_means *t*-test function from the ggpubr R package (Kassambara, 2020), weighted means permutation test and Kolmogorov-Smirnov Tests from the stats R package (R Core Team, 2021). FindMarkers from the Seurat R package was used to perform DE analysis between groups. For DE between exposure groups and controls at each Day after filtering out genes encoding ribosomal proteins (Figures 5H, 6A), thresholds were set to the following: min. pct = 0.2, min. diff.pct = -Inf, logfc. threshold = 0.35. Genes with an adjusted *p*-value <0.05 were considered significant. GO analysis was performed using the DEenrichRPlot function of the mixscape R package with the “GO_Biological_Process_2018″database with the following thresholds: logfc. threshold = 0.35, max genes = 500 (Supplementary Figures S5D–G).

### 2.10 Open-access web applications

Datasets can be browsed and visualized in open-access web applications at https://neuroomicsexplorer.medisin.uio.no.

Bulk RNA-seq and DNAm data: https://neuroomicsexplorer.medisin.uio.no/bulkCitNeuronalDiff. The bulkCitNeuronalDiff web application contains five tabs. “bulkCitNeuronalDiff app information” provides a short description of the methods used and a graphical abstract. “Explore GE results” allows the user to select a results file from the bulk differential gene expression analysis to explore, either comparisons between days in control cells, citalopram dose-response (DR) or citalopram time-response (TR) analysis. The results are viewed in a searchable data table in addition to a statistics histogram and volcano plots with gene information at each point. The user can filter data on significance level, download plots as PDF files and tables as CSV files. “Explore DNAm results” has the same functionality as “Explore GE results”, applied to the bulk differential DNAm analysis results. Here, only data with FDR < 0.05 is shown to improve speed of the application. “GE boxplots” and “DNAm boxplots” provides functionality to plot time- and dose-response plots for gene expression and DNAm data equivalent to those in Figures 2–4. Here, the user can search for any gene or CpG, select linear or non-linear line type, subset data on group, day, (citalopram) concentration or treatment, and download plots as PDF files.

Single-cell data: https://neuroomicsexplorer.medisin.uio.no/scRNACitNeuronalDiff. The scRNACitNeuronalDiff web application contains seven tabs, which provides functionality to visualize the scRNA data in different ways. “CellInfo vs GeneExp” allows the user to plot two principal component (PC), t-distributed Stochastic Neighbor Embedding (tSNE), or Uniform Manifold Approximation and Projection (UMAP) plots side-by-side, colored by various cell information metadata, such as original identity, day, cell cycle phase or different clustering parameters, as well as the gene expression of any selected gene. In the “CellInfo vs CellInfo” and “GeneExp vs GeneExp” tabs, the user can plot either two cell information plots or two gene expression plots side-by-side, respectively. In the “Gene coexpression” tab, co-expression of two selected genes can be plotted together in the same PCA, tSNE or UMAP. In the “Violinplot/Boxplot” tab, the user can plot cell information on the X-axis and cell information or gene expression of any selected gene on the Y-axis of a violin plot or boxplot. In the “Proportion plot” tab, the proportion or number of cells can be plotted for available cell information, and finally in the “Bubbleplot/Heatmap” tab gene expression in up to 50 genes can be plotted together in a bubbleplot or heatmap. In each tab, the user can subset data based on cell and all plots can be downloaded as PDF or PNG files.

### 2.11 Statistical analysis

Statistical analyses were performed in R version 4.2 (R Core Team, 2021) using edgeR (Robinson et al., 2010), Limma (Ritchie et al., 2015), Seurat (Stuart et al., 2019; Hao et al., 2024), ggpubr (Kassambara, 2020) and stats (R Core Team, 2021) packages. Details are described in the relevant methods above.

## 3 Results

### 3.1 Experimental set-up and validation of neuronal differentiation of hESCs

**FIGURE 1:**
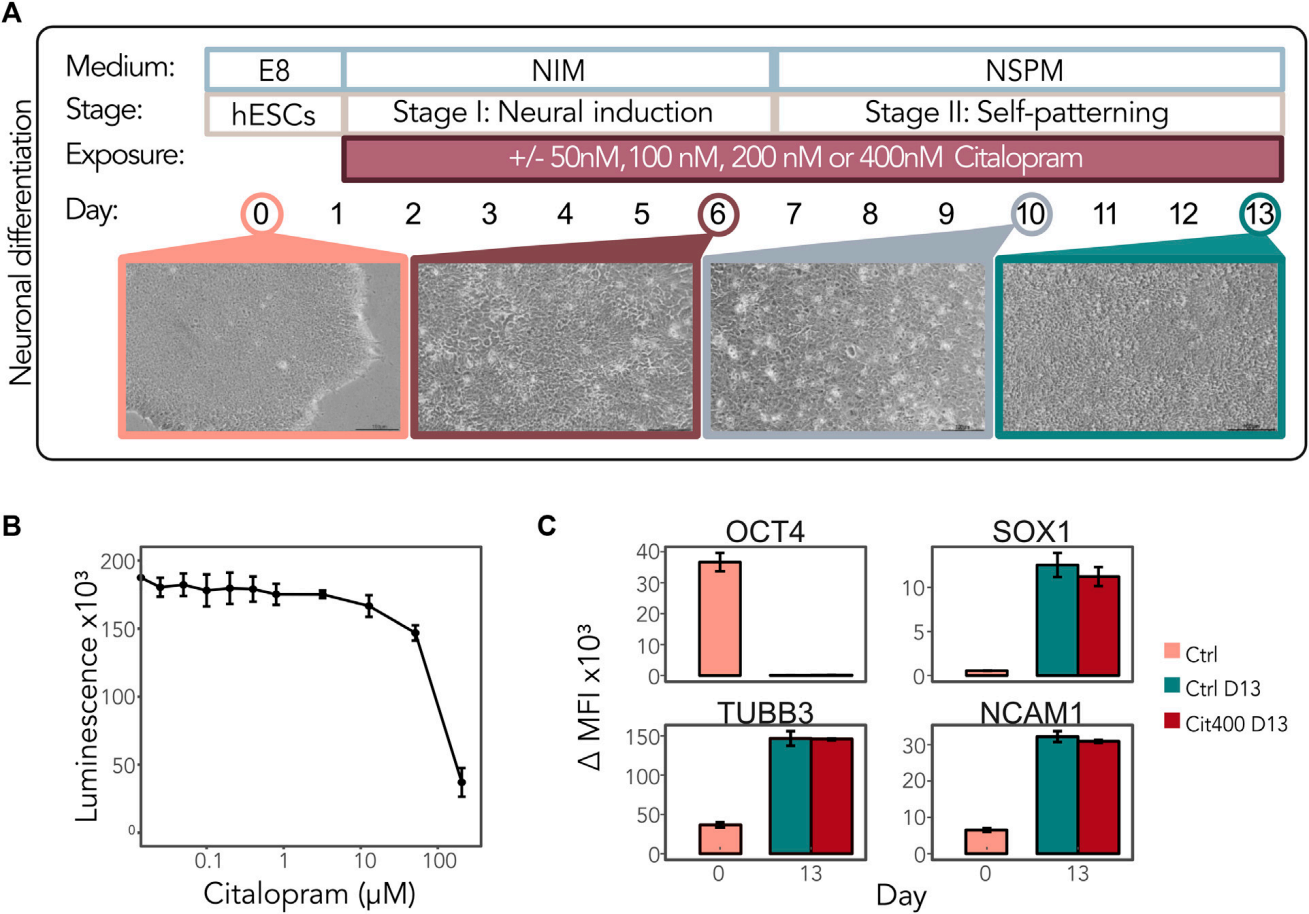
Neurodevelopmental effects of citalopram in a model of early human neuronal development. **(A)** Schematic representation of neuronal differentiation of hESCs. The cells were continuously exposed to media only (control cells) or 50, 100, 200 or 400 nM citalopram from Day 1 and throughout the differentiation process. Samples were collected for multi-omics analyses at Day 0, 6, 10 and 13. **(B)** Viability of hESCs after citalopram exposure for 24 h. **(C)** The presence of the stem cell marker OCT4 and neuronal markers SOX1, TUBB3 and NCAM1 was assessed by flow cytometry at Day 0 and Day 13 in control cells and cells exposed to 400 nM citalopram. ΔMFI, delta median fluorescence intensity.

We hypothesized that early exposure to citalopram affects DNAm and regulation of the genes involved in neuronal differentiation. To comprehensively study the effect of citalopram on the epigenetic and transcriptional profiles in a model of early human neurodevelopment, we used a neuronal differentiation protocol optimised for neurotoxicology studies recently published by our group (Samara et al., 2022a; 2022b) (Figure 1A). The hESCs were exposed to physiological concentrations of citalopram (50, 100, 200 and 400 nM, reflecting human therapeutic doses (Hendrick et al., 2003; Rampono et al., 2009; Paulzen et al., 2017; Hiemke et al., 2018)) from Day 1 and throughout differentiation to Day 13 to model the effect of daily maternal intake of citalopram on neurodevelopment in the first trimester. The selected concentrations of citalopram did not affect hESC viability after 24 h of exposure (Figure 1B). There were no visually detectable morphologic differences between cells exposed to citalopram and unexposed control cells at any stage (Supplementary Figure S1). Flow cytometric analysis confirmed that the transcription factor OCT4, involved in establishment and maintenance of pluripotency, was present at Day 0 and absent at Day 13 in both unexposed control cells and cells exposed to 400 nM (Cit400) citalopram (Figure 1C). The levels of neural progenitor marker SOX1 increased at Day 13 compared to Day 0. Similarly, the levels of tubulin beta class III (TUBB3), a structural cytoskeletal protein involved in neurogenesis, and cell adhesion protein NCAM1, a regulator of neurogenesis, neurite outgrowth and cell migration, increased at Day 13. Exposure to Cit400 did not affect the presence of these markers at Day 13.

To validate the neuronal differentiation process in the protocol, we performed differential gene expression and DNAm analyses in control samples between Day 0, Day 6, Day 10 and Day 13. These data have been made available in a web tool bulkCitNeuronaldiff, enabling browsing and visualization of differential analysis, gene expression and DNAm levels. As expected, major changes in gene expression and DNAm occurred during neuronal differentiation of the control cells (Supplementary Figures S2, S3 and bulkCitNeuronaldiff), and samples clustered according to Day (Supplementary Supplementary Figures S2A, B). To assess whether the differentiation was affected by the higher initial cell seeding and if differentiation corresponded with our group´s recently published data (Samara et al., 2022b), we identified overlapping differentially expressed genes (DEGs) and differentially methylated CpGs (DMCs) between Day 0 and 13 (Supplementary Figures S2C, D). Of the DEGs and DMCs identified in the present study, 83% and 75% overlapped with Samara and Spildrejorde et al. (Samara et al., 2022b), respectively. There were also many unique DEGs and DMCs in the present study, which could indicate some batch effects, either during differentiation, and/or at later steps. Volcano plots for gene expression and DNAm are shown in Supplementary Figures S2E, F and top DEGs heatmaps between Days are shown in Supplementary Figure S2G.

Many of the top shared biological processes (BPs) for both gene expression changes (Supplementary Figure S3A) and DNAm changes (Supplementary Figure S3B) during differentiation included relevant terms for neuronal differentiation, such as *developmental induction*, *neuron differentiation* and *nervous system development*. The DEGs overlapped with differentially methylated genes (DMGs) at the different stages (Supplementary Figures S3C–E), thus showing good correspondence between datasets. The number of DEGs and DMCs overlapping between stages are shown in Supplementary Figures S3F, G. Expression of pluripotency marker genes such as *POU5F1*, *NANOG* and *LIN28A* decreased after Day 0, whereas neuronal differentiation marker genes such as *OTX2*, *MAP2*, *NEUROD1*, *STMN2*, *TUBB3* and *FOXG1* increased during differentiation (Supplementary Figure S2H).

### 3.2 Citalopram affects gene expression and DNAm levels over time during neuronal differentiation

**FIGURE 2:**
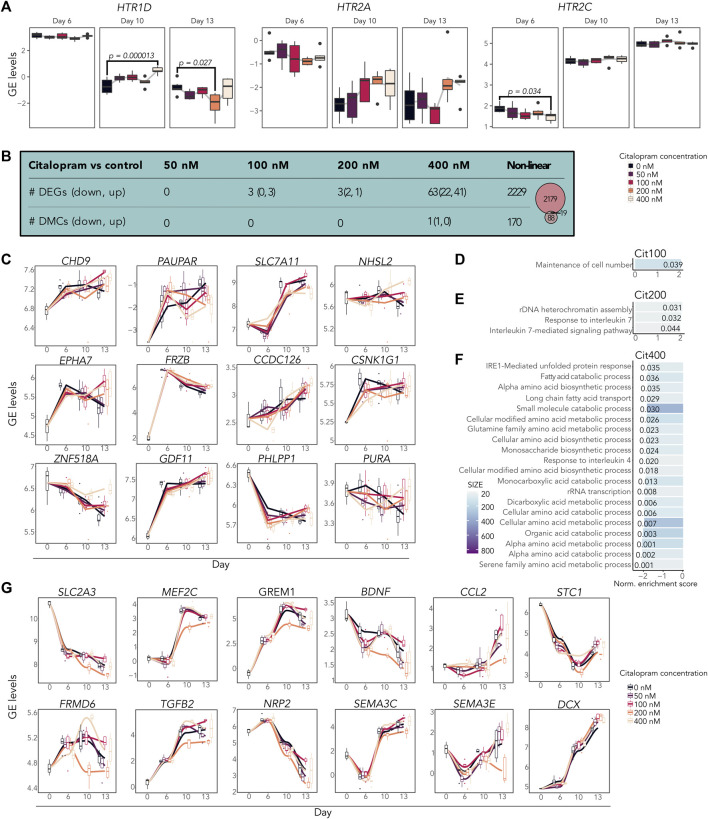
Time-response of citalopram-exposure during neuronal differentiation of hESCs. **(A)** Gene expression (GE) levels (log counts per million, logCPM) of selected serotonin receptor genes for Day 6 to Day 13. **(B)** Table showing the number of genes and CpGs that responded to the different concentrations of citalopram over time (Day 6 to Day 13). Venn diagram showing overlapping DEGs and DMGs. **(C)** GE levels (logCPM) of selected linear time-response DEGs. **(D–F)** Shared biological processes (BPs) among genes that were differentially expressed in cells exposed to **(D)** 100 nM, **(E)** 200 nM or **(F)** 400 nM citalopram over time. **(G)** GE levels (logCPM) of selected non-linear time-response DEGs. Genes with adjusted *p*-value <0.05 were considered significant.

Using bulk RNA-seq, we first examined the expression of serotonin receptors in both control and citalopram-exposed cells. Some of the genes encoding serotonin receptors were present at Day 0, albeit very lowly expressed throughout differentiation (*HRT1A*, *HRT1B*, *HRT1F*, *HRT3A* and *HRT7*; not shown). Expression of *HTR1D* and *HTR2A* peaked at Day 6 and decreased during differentiation, whereas expression of *HTR2C* increased during differentiation (Figure 2A), confirming the transcriptional presence of serotonin receptors.

To study whether exposure to citalopram caused gene expression or DNAm changes over time during neuronal differentiation of hESCs, we performed a linear time-response analysis and compared each concentration of citalopram from Day 6 to Day 13 to unexposed control cells using bulk omics data (Figure 2 and bulkCitNeuronaldiff). Only one position annotated to *RABEP2* was differentially methylated between 400 nM citalopram and control over time (Figure 2B and bulkCitNeuronaldiff). In contrast, we observed a dose-dependent linear effect of citalopram exposure on the number of DEGs (Figure 2B and bulkCitNeuronaldiff). Differentiating cells exposed to 50 nM citalopram (Cit50) did not show any differential expression compared to control over time (Figure 2B). However, exposure to 100 (Cit100) and 200 nM citalopram (Cit200) resulted in three DEGs, whereas Cit400 resulted in 63 DEGs.

Among the DEGs identified was the chromatin remodeler *CHD9*, which was upregulated in Cit100 cells compared to controls. *PAUPAR*, a gene encoding a long non-coding RNA that regulates *PAX6* (Vance et al., 2014), and *SLC7A11,* associated with ASD (Rojas-Charry et al., 2021), was significantly downregulated in Cit400 cells over time (Figure 2C). In contrast, *NHSL2* (associated with ASD (Gerges et al., 2022)), *EPHA7* (important for neuronal maturation and synaptic function (Clifford et al., 2014)), WNT antagonist *FRZB*, *CCDC126* (associated with depression (Gui et al., 2018)), *CSNK1G1* (involved in glutaminergic neurotransmission (Chergui et al., 2005))*,* transcriptional regulator *ZNF518A*, *GDF11* (a growth factor involved in neurogenesis and differentiation of neuronal subtypes (Shi and Liu, 2011)) and *PURA* (essential to neurodevelopment and linked to neuroprotection (Daniel and Johnson, 2018; Molitor et al., 2021)) were upregulated in Cit400 cells over time (Figure 2C). *PHLPP1* was differentially expressed over time in both Cit100, Cit200 and Cit400 compared to controls. Interestingly, *PHLPP1* is involved in many important functions in the nervous system, including memory formation, cellular survival and proliferation (Mallick et al., 2022). Further, we performed GSEA analysis to elucidate if the DEGs identified between citalopram-exposed and unexposed control cells shared any biological functions (Figures 2D–F). Results from this analysis revealed enrichment of different metabolic and catabolic processes, including fatty acid and amino acid biosynthetic processes among the DEGs.

Citalopram may also induce non-monotonic responses due to complex pharmacodynamic processes (Ludden, 1991). Using a non-linear regression model to identify time-dependent effects of citalopram, 2,229 genes were differentially expressed in citalopram-exposed cells at all concentrations compared to controls (Figure 2B). The DEGs included genes involved in neuronal development and brain function, such as *FRMD6* (involved in neuronal differentiation (Chen et al., 2021)), *SLC2A3* (a neural glucose transporter (Ziegler et al., 2020)), *GREM1* (antagonist of bone morphogenic protein (Ichinose et al., 2021)), *MEF2C* (important for neuronal differentiation and axogenesis (Zhang and Zhao, 2022)), *TGFB2* (essential for neurodevelopment (Meyers and Kessler, 2017)) and *DCX* (involved in neuronal migration (Ayanlaja et al., 2017)) (Figure 2G). Further, *NRP2*, *SEMA3C* and *SEMA3E,* involved in axon guidance during neurodevelopment (Oh and Gu, 2013)*,* were downregulated in Cit200. Some DEGs linked to depression and neuroinflammation (*BDNF* (Porter and O'Connor, 2022), *CCL2* (Curzytek and Leśkiewicz, 2021), *STC1* (Chao et al., 2021), *MEF2C* (Hyde et al., 2016)) were also downregulated in Cit200 over time (Figure 2G). The levels of the non-linear DEGs fluctuate with day and concentration, thus the biological interpretation is difficult. However, many of the DEGs are dysregulated in Cit200 compared to controls, suggesting that this concentration has a substantial effect on cells undergoing neuronal differentiation.

**FIGURE 3:**
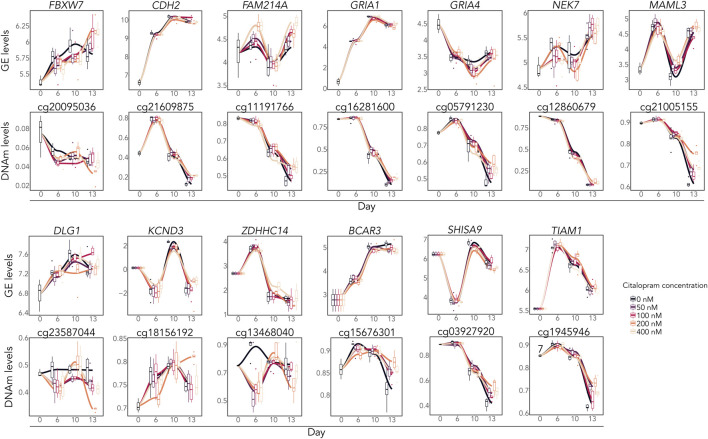
Overlapping differentially expressed and methylated time-response genes. Gene expression levels (logCPM) and DNAm levels (beta values) of selected overlapping DEGs and corresponding DMCs.

DNAm analysis showed that citalopram induced non-linear time-dependent changes at 170 CpGs, annotated to 107 genes. No significant gene ontology terms were identified. Of the 107 DMGs, 19 overlapped with DEGs (Figures 2B, 3). Some of the overlapping genes are involved in processes crucial for neurodevelopment (*FBXW7* (Yang et al., 2021), *CDH2* (László and Lele, 2022)), neuroinflammation (*NEK7* (Chen et al., 2019)), neuronal excitability (*ZDHHC14* (Sanders et al., 2020)), synaptic plasticity (*SHISA9* (Kunde et al., 2017)), neurite outgrowth (*TIAM1* (Ehler and Salinas, 1997)) and neurotransmission (*DLG1* (Marziali et al., 2019), voltage-gated potassium channel *KCND3*, glutamate receptors *GRIA1* and *GRIA4*) (Figure 3). Further, genes linked to mood disorders and stress response were identified (*BCAR3* (Han et al., 2020), *FAM214A* (Witte et al., 2022), *MAML3* (Kuehner et al., 2023)).

### 3.3 Citalopram exposure induces dose-dependent changes in gene expression during neuronal differentiation

**FIGURE 4:**
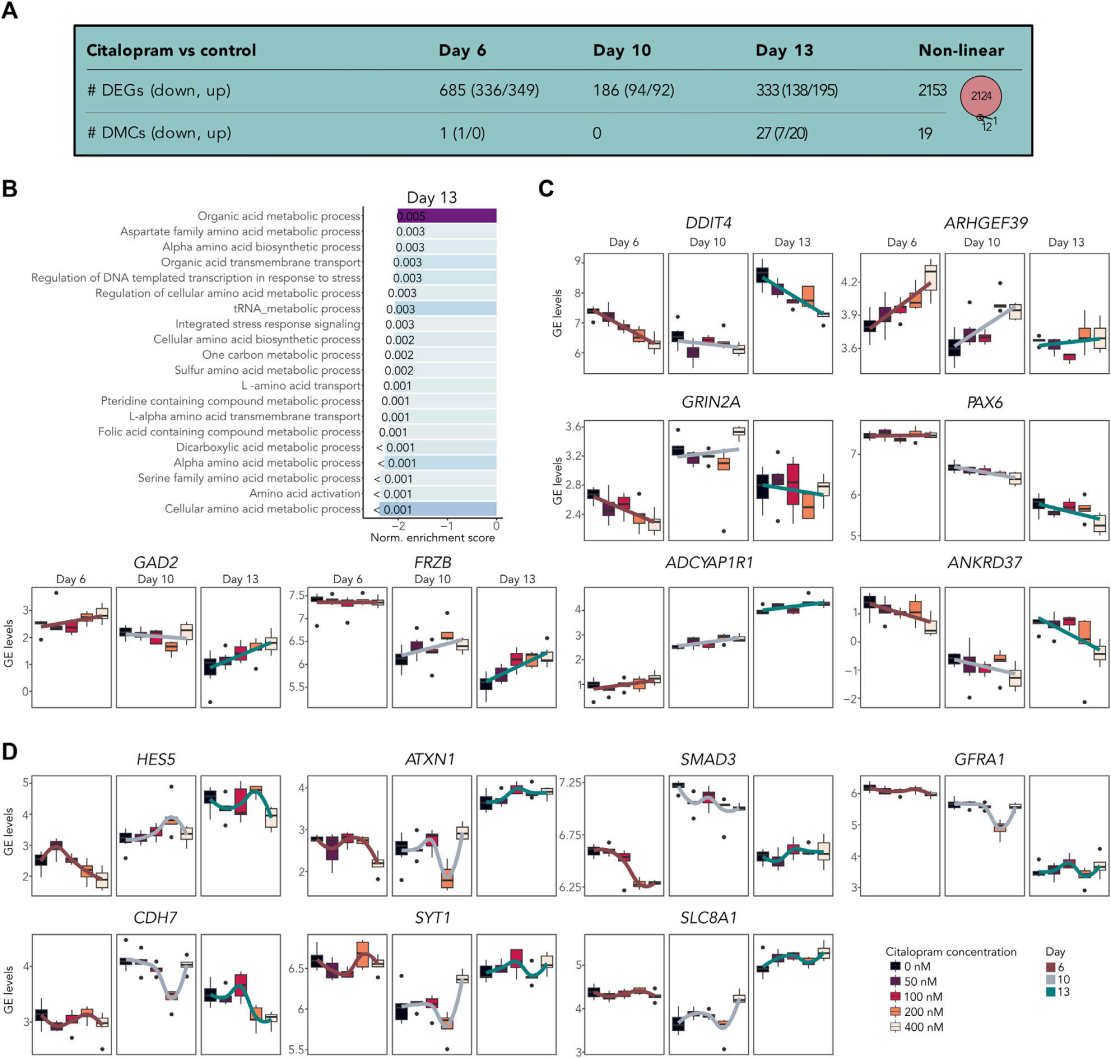
Citalopram-exposure affects gene expression in a dose-dependent manner during neuronal differentiation. **(A)** Number of DEGs and DMCs identified in the dose-response analysis. Venn diagram showing overlapping DEGs and DMGs. **(B)** Top shared BPs between genes that responded to citalopram in a dose-dependent manner at Day 13. **(C)** GE levels (logCPM) of selected linear dose-response DEGs. **(D)** GE levels (logCPM) of selected non-linear dose-response DEGs. Genes with adjusted *p*-value <0.05 were considered significant.

To assess the dosage-effect of citalopram, we performed a bulk RNA-seq dose-response analysis. To identify both potential monotonic and non-monotonic responses, we tested both the linear and non-linear effect of increasing citalopram concentrations compared to control cells (Figure 4 and bulkCitNeuronalDiff). Employing the linear regression model for each differentiation day we identified 685 DEGs associated with citalopram exposure in a dose-dependent manner compared to control cells at Day 6. At Day 10 and 13, we identified 186 and 333 DEGs, respectively compared to Day 6, which was baseline in this comparison (Figure 4A). GSEA did not identify any significant shared BPs for dose-response genes at Day 6 or Day 10. At Day 13, however, BPs related to metabolic and catabolic processes were enriched (Figure 4B). Interestingly, some of the linear dose-response DEGs have been implicated in transmission and plasticity (*GRIN2A* (Paoletti et al., 2013), *GAD2* (Pan, 2012)), depression and antidepressant effect (*DDIT4* (Wang et al., 2018), *GAD2* (Unschuld et al., 2009)) anxiety and stress responses (*ADCYAP1R1* (Oyola and Handa, 2017; Wang et al., 2021)), WNT signalling (*FRZB* (Mitsiadis et al., 2017)), neurogenesis (*ARHGEF39* (Anijs et al., 2022)) and hippocampal volume (*ANKRD37* (Xu et al., 2022)) (Figure 4C). Further, linear differential DNAm analysis identified one DMC at Day 6 (*RABEP2*), none at Day 10 and 27 at Day 13 (annotated to, e.g., *MECOM*, *GRIN1*, *SORCS2*; Figure 4A and bulkCitNeuronalDiff).

The non-linear dose-response analysis identified 2,153 DEGs (Figures 4A,D and bulkCitNeuronalDiff). Most of these were also identified in the non-linear time-response analysis (n = 1703). We identified DEGs implicated in cell state transitioning in neural progenitor (*HES5* (Manning et al., 2019)), neuronal differentiation (*CDH7* (Feng et al., 2017)), neurogenesis (*SMAD3* (Hiew et al., 2021)), synaptic plasticity (*GFRA1* (Bonafina et al., 2019)), synaptic transmission (*SYT1* (Riggs et al., 2022)), neuronal ion homeostasis (*SLC8A1* (Blaustein and Lederer, 1999)) and memory and learning (*ATX1* (Lu et al., 2017)). Further, citalopram induced non-linear time-dependent changes at 19 DMCs, annotated to 13 genes (Figure 4A and bulkCitNeuronalDiff). Of the DMGs, one overlapped with DEGs (*GRIA1*, Figure 3). Overall, genes involved in brain function were dose-dependently dysregulated in cells exposed to citalopram.

### 3.4 Effect of citalopram exposure at different stages during neuronal differentiation

**FIGURE 5:**
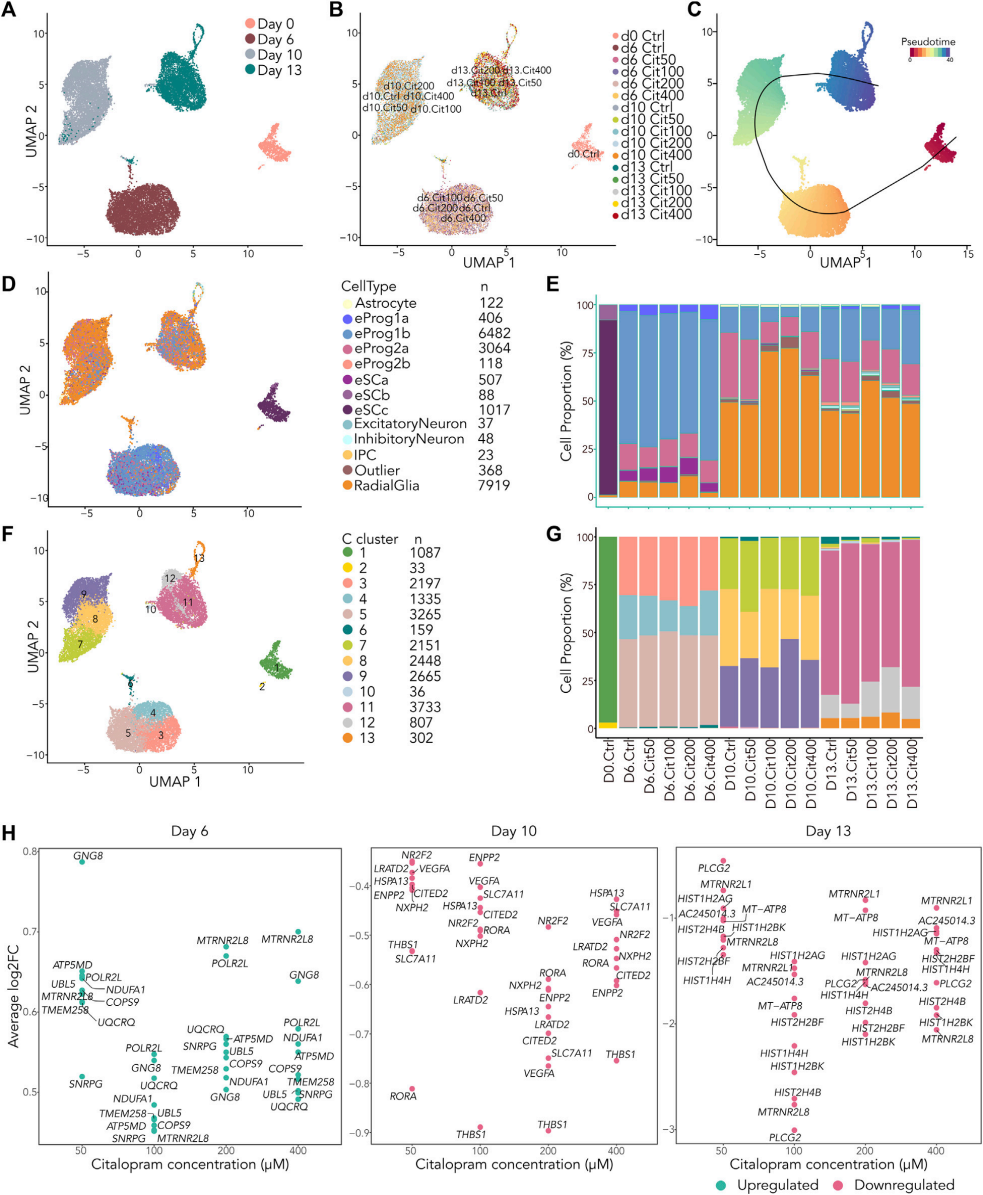
scRNAseq reveal small differences in cell type composition between cells exposed to citalopram and control cells. **(A–D)** All cells projected in UMAP plots colored by **(A)** Day, **(B)** exposure group, **(C)** slingshot pseudotime and **(D)** annotation to LaManno brain data cell types. **(E)** Proportion of cells in each exposure group annotated to cell types according to the LaManno brain and Bhaduri organoid datasets. eSCa-c; hESCs, eProg1-2; hESC-derived neuronal progenitor cells, IPC; intermediate progenitor cells. **(F)** UMAP plot of all cells colored by Seurat clusters and **(G)** corresponding proportion of cell of each exposure group annotated to clusters. **(H)** Single cell gene expression of top ten overlapping DEGs between cells exposed to different concentrations of citalopram compared to control cells at Day 6, Day 10 and Day 13.

In addition to the longitudinal and dose effects of citalopram exposure on gene expression and DNAm changes during differentiation, citalopram might exert more specific effects at different stages and within distinct cell-types. To identify dose-dependent cell-type specific gene expression signatures and potential alterations in cell type composition during differentiation, we also performed single-cell RNA sequencing (scRNAseq) at the four different timepoints (Day 0, 6, 10 and 13) (Figure 5 and Supplementary Figure S4). These data have been made available for browsing and visualization in a web tool “scRNA citalopram-exposure during neuronal differentiation of hESCs” (scRNACitNeuronalDiff).

After filtering of the data, a total of 20,217 cells were included in downstream analysis (Supplementary Figure S4A). As expected, cells clustered according to differentiation day (Figures 5A and Supplementary Figure S4C), whereas exposed and unexposed cells clustered together at each day (Figure 5B). The cells were ordered according to their pseudotime development using Slingshot (Street et al., 2018), confirming that pseudotime differentiation values increased from Day 0 to Day 13 (Figures 5C and Supplementary Figure S4D) reflecting progression through the differentiation process.

We used SingleR (Aran et al., 2019) and compared the cells to the scRNA La Manno brain dataset (LaManno et al., 2016) and the Bhaduri forebrain organoid dataset (Bhaduri et al., 2020). This revealed that most cells at Day 6 were classified as neuronal progenitors (LaManno et al., 2016), with some variation in the progenitor subtype depending on citalopram concentration (Figures 5D, E). At Day 10 and 13, most cells were classified as radial glia cells (Bhaduri et al., 2020), whereas a smaller proportion were classified as neuronal progenitors. In addition, at Day 13, a small proportion of cells were more differentiated, classified as intermediate progenitor cells, excitatory neurons and inhibitory neurons (Bhaduri et al., 2020). In control cells, expression of selected marker genes involved in neurodifferentiation at Day 13 were comparable with Day 13 in the Samara and Spildrejorde *et al.* study (Supplementary Figure S4E) (Samara et al., 2022b).

The cells resolved into 13 Seurat clusters at resolution 0.55 (Figures 5F, G and Supplementary Figures S4F–G), where pseudotime differentiation progressed from C1 to C11-13 (Supplementary Figure S4H). The top five DEGs for each cluster are visualized in Supplementary Figure S4I. Overall, these clusters and expression of genes confirm neuronal differentiation of the citalopram-exposed cells and control cells. There was some variation in cell proportions within the different clusters depending on exposure to citalopram or not. However, no specific dose- or time-dependent trend was found (Figure 5G).

Next, we investigated whether the scRNA dataset revealed any dose-dependent DEGs. These analyses identified overlapping DEGs between citalopram and control cells at all citalopram concentrations (Figure 5H). At Day 6, the top ten overlapping DEGs were upregulated in citalopram-exposed cells. Of these, genes involved in neuroinflammation (*COPS9* (Tian et al., 2023)), neuroprotection (*MTRNR2L8* (Karachaliou and Livaniou, 2023)) and mitochondrial function (*ATP5MD*, *NDUFA1*, *UQCRQ*) were identified. Interestingly, mitochondrial function is important for neurodevelopment, and dysfunction is known to play a crucial role in the pathogenesis of depression (Song et al., 2023). In contrast, at Day 10 and Day 13, the top ten overlapping DEGs were downregulated in citalopram-exposed cells. At Day 10, genes involved in neurodevelopment (*CITED2* (Wagner and MacDonald, 2021), *RORA* (Ribeiro and Sherrard, 2023), *NR2F2* (Tocco et al., 2021)), neuronal migration (*THBS1* (Blake et al., 2008)) and synaptic function (*VEGFA* (Licht et al., 2009)) were identified. Further, the glutamate transporter *SLC7A11* that was also identified in the bulk time-response analysis (Figure 2C), was downregulated at Day 10. At Day 13, genes involved in mitochondrial function (*MT-ATP8*), genes associated with neuroprotection (*MTRNR2L8*, *MTRNR2L1* (Karachaliou and Livaniou, 2023)), and genes encoding histone variants (*HIST2H2BF*, *HIST1H4H*, *HIST1H2BK, HIST1H2AG*)) were identified. Of note, *HIST1H2AG,* has previously been associated with depression (Tirozzi et al., 2023).

**FIGURE 6:**
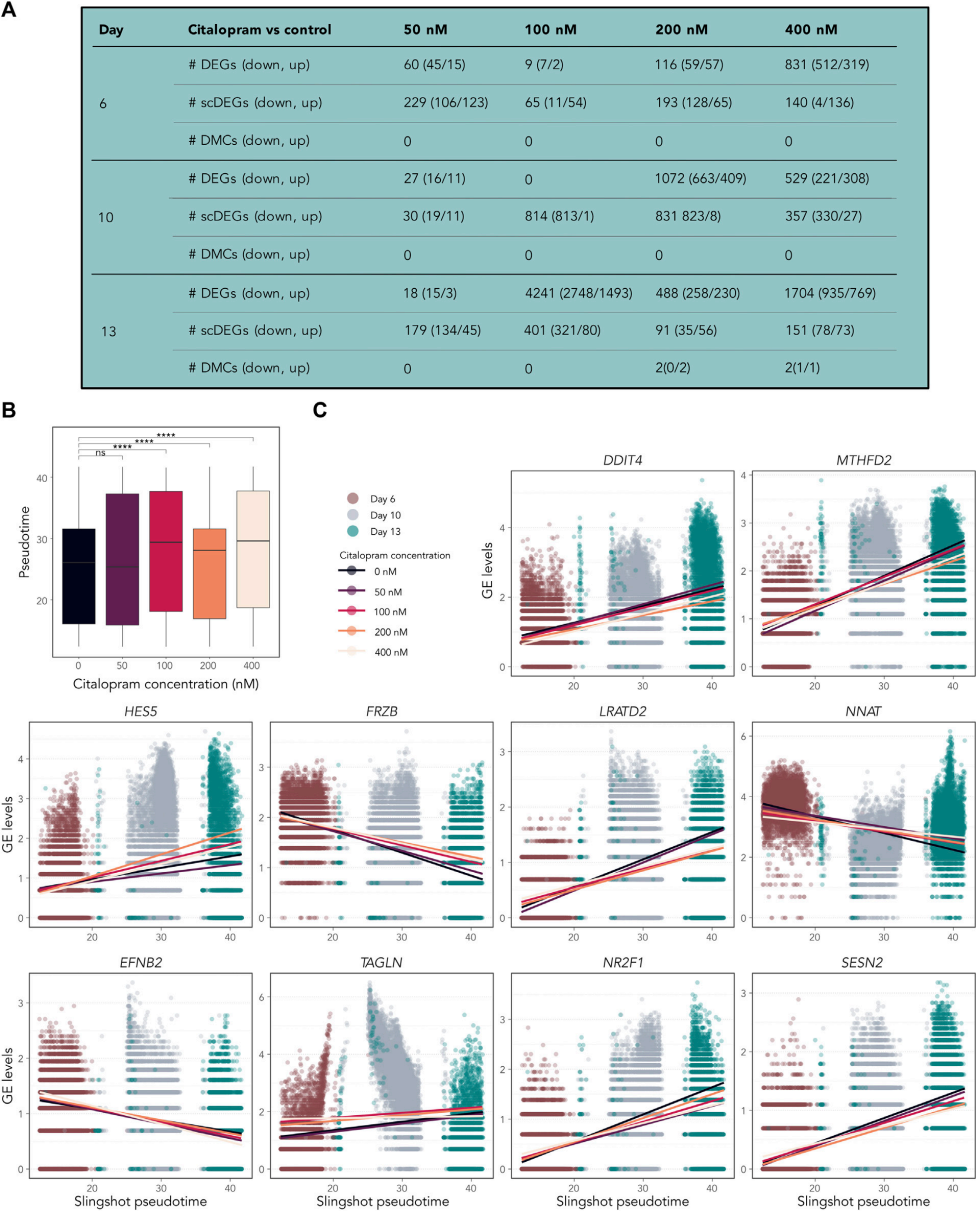
Temporally expressed genes respond differently in citalopram-exposed cells compared to control cells during differentiation. **(A)** Pairwise comparisons between citalopram and control for RNA-seq (DEGs), scRNAseq (scDEGs) and DNAm (DMCs) at Day 6–13. **(B)** Boxplot of Slingshot pseudotime values per exposure group for Day 6–13. Significant comparisons are marked with asterisks (Student’s t-test, ****: p ≤ 0.0001). **(C)** GE levels (logcounts) of *DDIT4*, *MTHFD2*, *HES5*, *FRZB*, *LRATD2*, *NNAT*, *EFNB2*, *TAGLN*, *NR2F1* and *SESN2* selected from the top 100 temporally expressed genes, which responded differently in citalopram-exposed and control cells across Slingshot pseudotime at Day 6–13. Each dot represents one cell, and the lines represent the average GE for each citalopram concentration across pseudotime.

We also performed pairwise differential analyses of bulk RNA-seq, scRNAseq and DNAm datasets to identify gene expression and DNAm differences between each concentration of citalopram compared to control at Day 6, 10 and 13 (Figure 6A and bulkCitNeuronalDiff). Four DMCs were identified in the DNAm analysis, suggesting that each dose of citalopram appears to have minor effect on DNAm at each neuronal differentiation stage (bulkCitNeuronalDiff). Cit200 had increased DNAm at CpGs annotated to *EYS* and *LAT* compared to control. Cit400 had decreased and increased DNAm at CpGs annotated to *DGKA* and *SLC30A8* compared to control, respectively. However, the expression of these genes was not changed at Day 13.

We identified varying numbers of DEGs at each Day between control and citalopram in both bulk and scRNAseq data (Figure 6A and bulkCitNeuronalDiff), some of which were specific for citalopram and controls, whereas others overlap between several citalopram concentrations (Supplementary Figures S5A–C). Furthermore, similar GO terms related to mitochondrial function, and different metabolic and catabolic processes (Supplementary Figure S5D) were common among the top enriched BPs between different comparisons, in the scRNA analyses. At Day 10, downregulated terms involved in regulation of transcription and stress response were identified (Supplementary Figure S5D). At Day 13, more BPs involved in chromatin organization, catabolic and metabolic processes were enriched, in addition to upregulation of *central nervous system development*, *axon development* and *generation of neurons* in Cit200 cells compared to controls.

### 3.5 Citalopram enhances differentiation

To investigate if citalopram induced changes in the pseudotemporal ordering of the cells, differential topology analysis of the slingshot trajectory was performed. After removing Day 0 from the dataset, a subtle but significant difference in pseudotime differentiation was observed between citalopram-exposed cells and control cells (Figure 6B). Specifically, citalopram-exposed cells had higher mean pseudotime levels compared to control cells, irrespective of differentiation day. This result was confirmed using a more robust permutation test (Supplementary Figure S6A) and Kolmogorov-Smirnov test (*p*-values 5.5 × 10^−4^, <2.2 × 10^−16^, 2.2 × 10^−16^, <2.2 × 10^−16^, for 50, 100, 200 and 400 nM citalopram compared to control cells, respectively). These results suggest that citalopram exposure subtly enhances neuronal differentiation of hESCs.

Some temporally expressed genes (Supplementary Figure S6B) responded differently to citalopram exposure compared to control cells (Figure 6C and Supplementary Figure S6C). For *CHAC1*, *LRATD2*, *ENFB2*, *TAGLN*, *NE2F1*, *SESN2, TPM1, CARS*, *VEGFA*, *XBP1*, *DDIT4* and *HCRT* expression was decreased in citalopram-exposed cells compared to control cells in more differentiated cells. In contrast, *HES5*, *FRZB* and *NKX2-1* expression was increased in citalopram-exposed cells compared to more differentiated control cells. Further, *NNAT,* which is involved in intracellular signalling critical for differentiation, synaptogenesis and plasticity (Lin et al., 2010; Oyang et al., 2011) was upregulated in citalopram-exposed cells. Interestingly, *LRATD2* and *NNAT* have previously been linked to electroconvulsive therapy response in depressed patients (Sirignano et al., 2021). The scRNA pseudotime analysis identified genes also found in the bulk RNA-seq dose-response analysis (Figures 4C, D), such as *DDIT4*, *HES5* and *FRZB* (Figure 6C).

## 4 Discussion

We aimed to investigate the impact of maternal citalopram use in early embryonic neurodevelopment using a neuronal differentiation model, which has been optimized for neuropharmacology studies (Samara et al., 2022a; 2022b). The hESCs were exposed to therapeutic concentrations of citalopram from differentiation Day 1–13. The results presented in this study provide valuable insights into the effects of citalopram exposure on gene expression and DNAm during the initial stages of neuronal differentiation. To our knowledge, this is the first multi-omics analysis of citalopram-exposed differentiating hESCs.

The analyses of gene expression and DNAm changes during neuronal differentiation revealed significant changes in the unexposed control cells, consistent with the expected patterns of neuronal differentiation. Many DEGs and DMCs were associated with BPs related to neuronal differentiation and nervous system development and overlapped with the study by Samara and Spildrejorde et al. (Samara et al., 2022b). However, there were also unique DEGs and DMCs to this study, highlighting the importance of using internal controls for each neurotoxicology experiment. Taken together, these results confirmed neuronal differentiation, and the Day 6 and Day 10 cells adds to the knowledge of temporally waves of gene expression during the neural rosette stage and self-pattering phase of neuronal differentiation (Samara et al., 2022b). Further, serotonin receptors, implicated in depression aetiology and SSRI response (Nautiyal and Hen, 2017), were expressed in both controls and citalopram-exposed cells, suggesting that the cells in the neuronal differentiation model have potential to respond to serotonin signalling. However, serotonin levels were not measured, and cells did not express the serotonin transporter *SLC6A4*. Thus, studying citalopram exposure in this model may reflect indirect effects.

We identified citalopram-induced time- and dose-response effects on the expression of specific genes involved in neurodevelopment, neuronal migration, axon guidance, neuronal maturation, synaptic transmission, cell state transitioning and stress-response, which provides important insights into the molecular mechanisms underlying the potential effects of citalopram exposure on early brain development. Dysregulation of such crucial genes may potentially have an impact on cognitive and behavioural processes.

Interestingly, we also identified citalopram-induced time- and dose-response effects on the expression of genes associated with depression aetiology and potential therapeutic mechanisms. For example, *CCL2* (*MCP-1*), a chemokine involved in a range of neurobiological processes, has been associated with depression brain-immune system communication and suggested as a potential antidepressant target (Curzytek and Leśkiewicz, 2021). Similarly, *BDNF*, a growth factor involved in neuroinflammation, has been indicated in depression pathogenesis and antidepressant efficacy (Porter and O'Connor, 2022). Also, *STC1* has been shown to decrease neuroinflammation and attenuate depression-like symptoms in rats (Chao et al., 2021). These findings suggests that citalopram may influence key pathways and processes implicated in depression, such as neuroplasticity, neuroinflammation, cellular stress response and neurotransmitter regulation (Saveanu and Nemeroff, 2012).

The time- and dose-response analysis of bulk RNA revealed differentially expressed genes in citalopram-exposed cells compared to control cells, sharing BPs involved in amino acid metabolic and catabolic processes. This result is in line with previous studies where changes in plasma amino acids have been identified as response to SSRI treatment (Kaddurah-Daouk et al., 2013; Woo et al., 2015), also evident for (es)citalopram (MahmoudianDehkordi et al., 2021). In addition to being building blocks in biosynthetic and metabolic processes, amino acids are also involved with synaptic neurotransmission. Understanding how antidepressants affect these may aid the understanding of depression aetiology and treatment response.

Hippocampal impairment has been associated with major depression disorder (MacQueen and Frodl, 2010). Citalopram exposure induced changes in *ANKRD37* and *GDF11*, important for hippocampal volume (Xu et al., 2022; Moigneu et al., 2023), Interestingly, in mice, infusion of GDF11 enhanced hippocampal neurogenesis and attenuated depression-like symptoms (Moigneu et al., 2023). Further, a causal negative correlation between *ANKRD37* expression and hippocampal volume has been previously identified (Xu et al., 2022). The present study identified increased expression of *GDF11* and decreased expression of *ANKRD37*, suggesting that citalopram may potentially mediate its therapeutic action though hippocampal recovery. This result is consistent with several previous studies, where antidepressants have been reported to enhance hippocampal neurogenesis (Malberg et al., 2000; Anacker et al., 2011; Perera et al., 2011; Mateus-Pinheiro et al., 2013).

We also identified genes associated with NDDs such as ASD (e.g., *SLC7A11, NHSL2, PAX6, CDH2*, *ATX1*) and ADHD (e.g., *CDH2*), suggesting a potential link between citalopram exposure and the molecular pathways involved in the pathogenesis of NDDs. For example, altered expression of *SLC7A11* may disrupt amino acid transport and redox balance, which have been implicated in ASD pathogenesis (Rojas-Charry et al., 2021). Further, dysregulation of *CDH2* may disrupt synaptic function, indicated in ASD (László and Lele, 2022). Overall, the time- and dose-response results indicate that citalopram affects molecular mechanisms important during fetal neurodevelopment. In contrast, the corresponding DNAm analysis identified relatively few significant changes. We identified 19 genes with changes in both gene expression and DNAm, indicating that the citalopram-induced gene expression changes were modulated by other mechanisms other than DNAm, by CpGs not covered by the EPIC array, or by CpGs which we did not have enough power to detect.

To gain a more detailed understanding of the effects of citalopram exposure at different stages of neuronal differentiation and within specific cell-types, scRNA-seq was performed. The analysis identified distinct cell clusters corresponding to the different stages of differentiation. The scRNA-seq analysis revealed dose-dependent cell-type specific gene expression signatures. For example, citalopram exposure increased expression of *FRZB,* involved in WNT signalling (Mitsiadis et al., 2017)*.* The citalopram-induced increase in *FRZB* expression was identified in both bulk RNA-seq time- and dose-response analysis and in scRNA-seq pseudotime analysis. Interestingly, *FRZB*, was downregulated in paracetamol-exposed cells using the same neuronal differentiation model, albeit at different time points (Spildrejorde et al., 2023), indicating that the WNT-pathway respond differently to citalopram compared to paracetamol. Another gene that was differentially expressed in both paracetamol-exposed cells and citalopram-exposed cells was neuronal transcription factor *PAX6*. In paracetamol-exposed cells *PAX6* was upregulated, indicating a delay in neuronal differentiation (Spildrejorde et al., 2023). In contrast, *PAX6* expression was downregulated in citalopram-exposed cells in a dose-dependent manner, suggesting that citalopram may enhance neuronal differentiation. This is also in line with the pseudotemporal analysis, showing that citalopram-exposed cells were more differentiated compared to control cells. Interestingly, the SSRI sertraline has also shown to increase *DCX* expression indicating enhanced neuronal differentiation (Anacker et al., 2011), consistent with the citalopram-induced increase in *DCX* at Day 10 and 13.

There are several limiting factors to this study. The complexity of neurodevelopment in the human brain cannot fully be recapitulated using a simplified *in vitro* hESC neuronal differentiation model. We could not account for fetal-fetal or maternal-fetal signalling interactions. Additionally, the study only examined the effects of citalopram during a narrow time window of neuronal differentiation and at selected concentrations. Using one hESC line and no phenotype model, exploring genetic susceptibility related to disease risk was beyond the scope of this study. Furthermore, the model may not be suitable to investigate citalopram therapeutic mechanism of action, thus the interpretation in that direction remain speculative. Further research is needed to delineate the effects of citalopram exposure at different stages of neurodevelopment and to investigate potential long-term consequences.

To comply with the Findability, Accessibility, Interoperability and Reusability (FAIR) principles, we share both bulk RNA-seq and DNAm (bulkCitNeuronalDiff) and scRNAseq data (scRNACitNeuronalDiff) in open-access user-friendly web applications. These provide interactive functionality for browsing results, visualising genes and CpGs, and downloading figures and tables, allowing the reader to explore the data easily, fostering transparency, reproducibility, and collaboration in scientific research.

There are several translational aspects of the results obtained from this protocol and findings in, for example, cord blood from children prenatally exposed to maternal citalopram use. First, it can provide mechanistic insights into how citalopram exposure may influence DNAm and gene expression patterns during neuronal development, helping researchers understand the potential molecular pathways involved. Second, it can generate hypotheses for further investigation in cord blood studies, suggesting specific genes that may be altered in infants exposed to maternal citalopram use. Third, comparative analysis with findings in cord blood can identify overlapping genes and support a potential link between citalopram exposure, omics modifications and neurodevelopmental outcomes. Such findings could strengthen causal inference and clinical translation of findings in cord blood on early brain development. Last, it can serve as a potential basis for validating and replicating cord blood findings.

In conclusion, this study provides important insights into the effects of early citalopram exposure on gene expression and DNAm during neuronal differentiation. The findings highlight the time- and dose-dependent alterations in gene expression associated with neurodevelopment, axon guidance, neuronal maturation, synaptic transmission and depression. The study’s multi-omics approach offers valuable mechanistic insights and potential translational implications. Overall, this study contributes to our understanding of citalopram’s impact on early brain development and provides a basis for future investigations.

## Data Availability

The data presented in the study are deposited in the NCBI’s GEO repository, accession number GSE260892 (Subseries RNA-seq: GSE260888, DNAm: GSE260890, scRNA-seq: GSE260889).
