# Supplementary material for: Citalopram exposure of hESCs during neuronal differentiation identifies dysregulated genes involved in neurodevelopment and depression
Source: Front Cell Dev Biol. 2024 Jul 11;12:1428538. doi: 10.3389/fcell.2024.1428538 (PMC11269147; doi:10.3389/fcell.2024.1428538)
Supplement: Supplementary file 1 [file DataSheet1.PDF]

Supplementary Material

Table S1. Overview over reagents and resources.

REAGENT or RESOURCE	SOURCE	IDENTIFIER
Antibodies		
PE Mouse Anti-Human NCAM-1 (CD56)	BD Biosciences	CAT#563238; RRID:AB_2738087
PE Mouse IgG1, κ Isotype Control	BD Biosciences	551436
PE Mouse anti-Human Sox1	BD Biosciences	561592; RRID:AB_10714631
PE Mouse IgG1, κ Isotype Control	BD Biosciences	551436
Alexa Fluor® 488 Mouse anti-β-Tubulin, Class III	BD Biosciences	560381; RRID:AB_1645344
Alexa Fluor®488 Mouse IgG2a, κ Isotype control	BD Biosciences	558055; RRID:AB_1645612
PE Mouse anti-Oct3/4	BD Biosciences	560186; RRID:AB_1645331
PE Mouse IgG1, κ Isotype Control	BD Biosciences	551436
Chemicals, peptides, and recombinant proteins		
Geltrex™ LDEV-Free, hESC-Qualified, Reduced Growth Factor Basement Membrane Matrix	ThermoFisher	A1413302
KnockOut™ DMEM	ThermoFisher	10829018
PBS, no calcium, no magnesium	ThermoFisher / GIBCO	14190
Dimethyl-sulfoxide, DMSO	Sigma-Aldrich/ Merck	D8418
Accutase™ Cell Detachment Solution	STEMCELL Technologies	7920
UltraPure 0.5 M EDTA, pH 8.0	ThermoFisher	15575020
Citalopram	Sigma-Aldrich	C7861-10MG
RHO/ROCK Pathway Inhibitor Y-27632	STEMCELL Technologies	SCM075
Essential 8™ Medium	ThermoFisher	A1517001
Poly-L-ornithine hydrobromide	Sigma-Aldrich/ Merck	P3655
Fibronectin (Bovine Protein, Plasma)	ThermoFisher	33010018
N2 supplement (100X)	ThermoFisher	17502048
Advanced DMEM/F-12	ThermoFisher	12634028
GlutaMAX™ Supplement	GIBCO/ ThermoFisher	35050061
Penicillin Streptomycin (10,000 U/mL)	ThermoFisher	15140122
LDN-193189	STEMCELL Technologies	72148
SB 431542 (hydrate)	Sigma-Aldrich / Merck	S4317
XAV939	STEMCELL Technologies	72674
B-27™ Supplement (50X), serum free	ThermoFisher	17504044
Cytofix Fixation Buffer	BD Biosciences	554655
1X Perm/Wash buffer	BD Biosciences	554723
Stain Buffer (FBS)	BD Biosciences	554656
Critical commercial assays		
Countess™ Cell Counting Chamber Slides	ThermoFisher	C10312
CellTiter-Glo® Luminescent Cell Viability Assay	Promega	G7570

RNA/DNA purification kit	Norgen Biotek Corp.	298-48700
RNase-Free DNase I Kit	Norgen Biotek Corp.	298-25720
Qubit™ RNA BR Assay Kit	ThermoFisher/Invitrogen	Q10211
Agilent RNA 6000 Nano Reagents	Agilent Technologies	5067-1512
Genomic DNA ScreenTape	Agilent Technologies	5067-5365
Genomic DNA Reagents	Agilent Technologies	5067-5366
TruSeq Stranded mRNA Library Prep Kit	Illumina	20020595
NovaSeq 6000 S4 Reagent Kit v1.5 (300 cycles)	Illumina	20028312
Infinium MethylationEPIC BeadChip Kit v.1.0_B3 (96 samples)	Illumina	WG-317-1003
30 mm MACS SmartStrainers	Miltenyi Biotech	130-110-915
Chromium Next GEM Single Cell 3′ Kit v3.1	10x Genomics	1000268
Chromium Next GEM Chip G	10x Genomics	1000120
Dual Index Kit TT Set A	10x Genomics	1000215
NovaSeq 6000 S1 Reagent Kit v1.5 (200 cycles)	Illumina	20028318
Deposited data		
RNA-seq, DNAm and scRNA-seq		NCBI GEO GSE260892 (Subseries RNA-seq: GSE260888, DNAm: GSE260890, scRNA-seq: GSE260889)
Experimental models: Cell lines		
Human embryonic cells, HS360, 46XY	Stockholms Medicinska Biobank / Sweden	HS360
Software and algorithms		
BBMap	(Bushnell, 2014)	https://jgi.doe.gov/data-and-tools/software-tools/bbtools/bb-tools-user-guide/bbmap-guide/
HISAT2	(Kim et al., 2015)	https://ccb.jhu.edu/software/hisat/index.shtml
featureCounts	(Liao et al., 2014)	https://subread.sourceforge.net/featureCounts.html
R Programming language	(R Core Team, 2019)	https://www.r-project.org/
Seurat Version 5	(Stuart et al., 2019; Hao et al., 2024)	https://github.com/satijalab/seurat
ShinyCell	(Ouyang et al., 2021)	https://github.com/SGDDNB/ShinyCell
Shiny	(Chang et al., 2023)	https://www.rstudio.com/products/shiny/
Slingshot	(Street et al., 2018)	https://www.bioconductor.org/packages/release/bioc/html/slingshot.html
10x Genomics Cell Ranger -Count	10x genomics	https://www.10xgenomics.com
BSgenome.Hsapiens.UCSC.hg38	DOI:10.18129/B9.bioc.BSgenome.Hsapiens.UCSC.hg38 (The Bioconductor Dev Team, 2023)(Team, 2023)	https://bioconductor.org/packages/release/data/annotation/html/BSgenome.Hsapiens.UCSC.hg38.html
EnsDb.Hsapiens.v86	DOI:10.18129/B9.bioc.EnsDb.Hsapiens.v86 (Rainer, 2017)	https://bioconductor.org/packages/release/data/annotation/html/EnsDb.Hsapiens.v86.html
clustree	(Zappia and Oshlack, 2018)	https://cran.r-project.org/web/packages/clustree/vignettes/clustree.html#references
scater	(McCarthy et al., 2017)	https://bioconductor.org/packages/release/bioc/html/scater.html
EdgeR	(Robinson et al., 2010)	https://bioconductor.org/packages/release/bioc/html/edgeR.html
GSEA	(Subramanian et al., 2005)	https://www.gsea-msigdb.org/gsea/index.jsp
Minfi	(Aryee et al., 2014)	https://www.bioconductor.org/packages/release/bioc/html/minfi.html

Limma	(Ritchie et al., 2015)	https://bioconductor.org/packages/release/bioc/html/limma.html
missMethyl	(Phipson et al., 2016)	https://bioconductor.org/packages/release/bioc/html/missMethyl.html
SingleR	(Aran et al., 2019)	https://github.com/dviraran/SingleR and https://bioconductor.org/books/release/SingleRBook/sc-mode.html
Single Cell Experiment	(Amezquita et al., 2020)	https://bioconductor.org/packages/release/bioc/html/SingleCellExperiment.html
viridis	(Garnier et al., 2023)	https://cran.r-project.org/web/packages/viridis/index.html
ggplot2	(Wickham, 2016)	https://cran.r-project.org/web/packages/ggplot2/index.html
tidyverse	(Wickham et al., 2019)	https://www.tidyverse.org/packages/
ggpubr	(Kassambara, 2020)	https://cran.r-project.org/web/packages/ggpubr/index.html
pheatmap	(Kolde, 2019)	https://CRAN.R-project.org/package=pheatmap
IlluminaHumanMethylationEPICmanifest	(Hansen, 2016)	https://bioconductor.org/packages/release/data/annotation/html/IlluminaHumanMethylationEPICmanifest.html
IlluminaHumanMethylationEPICanno.ilm10b5.hg38	EPIC annotation 1.0 B5	https://github.com/achilleasNP/IlluminaHumanMethylationEPICanno.ilm10b5.hg38
Rstudio	RStudio Team	https://www.rstudio.com/
Shiny tools for visualisation of datasets.	This paper	Bulk RNA-seq and DNAm data: https://neuroomicsexplorer.medisin.uio.no/bulkCitNeuronalDiff<br>Single-cell data: https://neuroomicsexplorer.medisin.uio.no/scRNACitNeuronalDiff

Table S2. FACS antibodies and final concentrations.

Antibody	Concentration (µg/100 µL)
Unstained	0
PE Mouse Anti-Human NCAM-1 (CD56)	0.0625
PE Mouse IgG1, κ Isotype Control	0.0625
PE Mouse anti-Human Sox1	0.12
PE Mouse IgG1, κ Isotype Control	0.12
Alexa Fluor® 488 Mouse anti-β-Tubulin, Class III	0.25
Alexa Fluor®488 Mouse IgG2a, κ Isotype control	0.25
PE Mouse anti-Oct3/4	0.25
PE Mouse IgG1, κ Isotype Control	0.25

Table S3. Overview over datasets.

	Day 0	Day 6					Day 10					Day 13					Total
Citalopram concentration (nM)	0	0	50	100	200	400	0	50	100	200	400	0	50	100	200	400	
Number of technical replicates																	
Bulk RNA-seq	3	6	6	6	6	6	6	6	5	5	6	6	6	5	5	6	89
Bulk DNAm	3	6	6	6	6	6	6	6	5	5	6	6	6	5	5	6	89
scRNA-seq (number of cells)	1120	1570	1493	1223	1602	964	1008	791	1994	1834	1623	830	823	1307	894	1141	20217

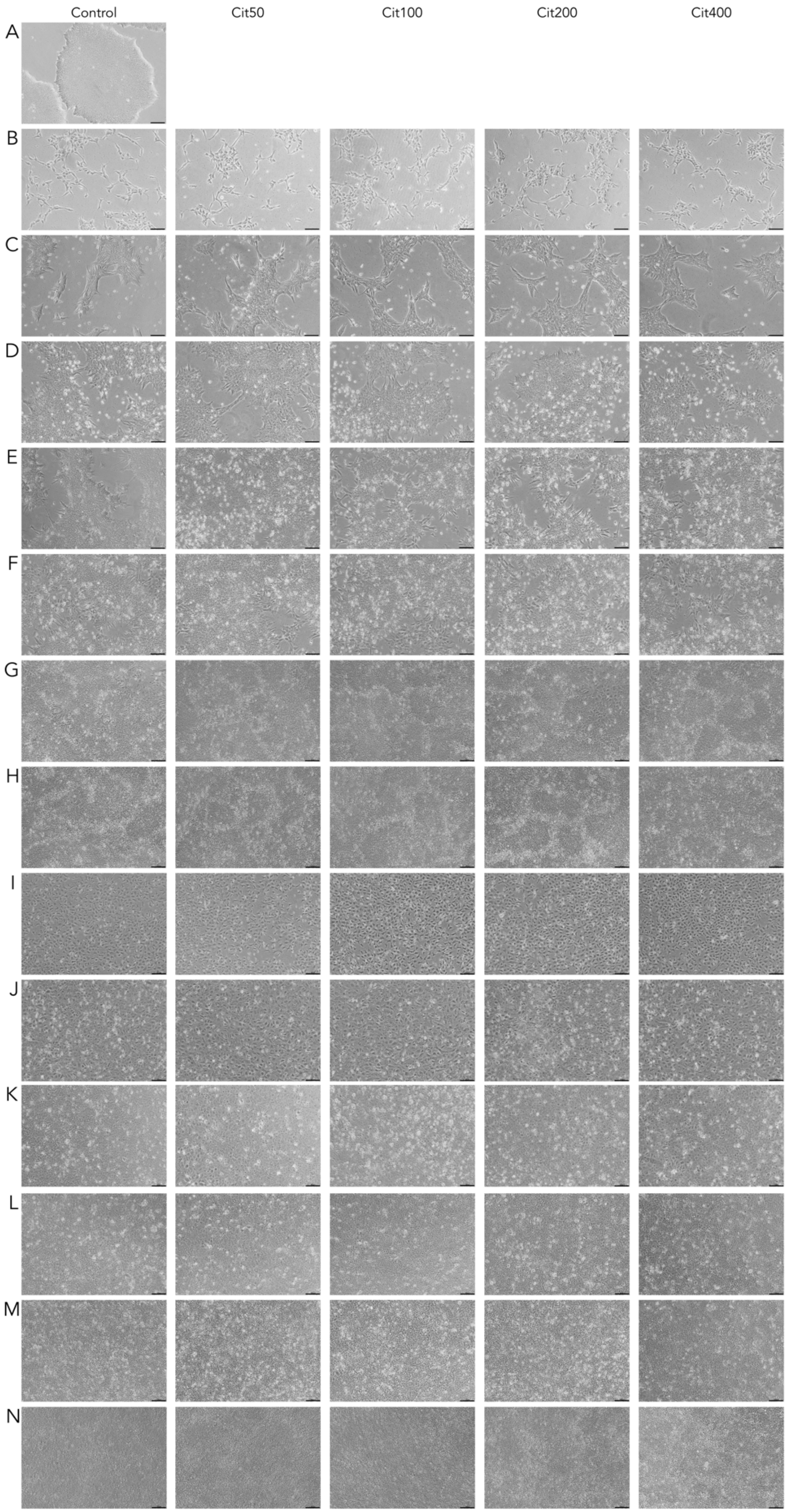

Figure S1. Timeline of differentiation, related to Figure 1. Brightfield images of the differentiating cells at A) Day 0, B) Day 1, C) Day 2, D) Day 3, E) Day 4, F) Day 5, G) Day 6, H) Day 7, I) Day 8, J) Day 9, K) Day 10, L) Day 11, M) Day 12 and M) Day 13. Scale bar corresponds to 100 µm.

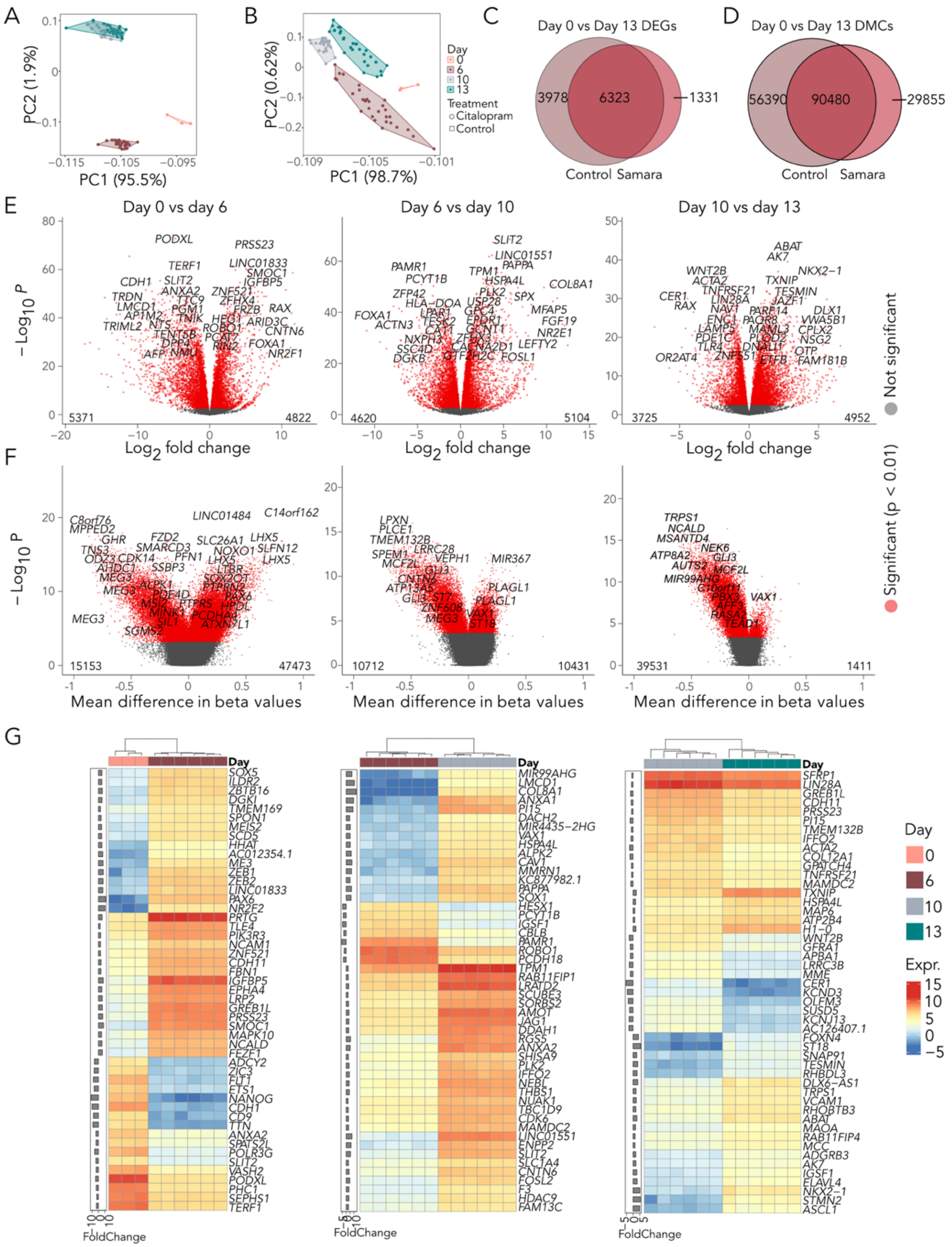

Figure S2. Gene expression and DNAm changes during neuronal differentiation in control cells. Principal component analysis of A) RNA-seq data, B) DNAm data. C-D) Venn diagrams showing the number of overlapping C) DEGs and D) DMCs between the current study´s control samples and

Samara et.al. 2022 (Samara et al., 2022)between Day 0 and 13. E-F) Vulcano plots showing E) DEGs F) DMCs between Day 0 and Day 6 (left), Day 6 and Day 10 (middle) and Day 10 and Day 13 (right). G) Expression levels visualized in heatmaps of the top 50 DEGs between Day 0 and Day 6 (left), Day 6 and Day 10 (middle) and Day 10 and Day 13 (right). Genes or CpGs with adjusted p-value < 0.01 were considered significant.

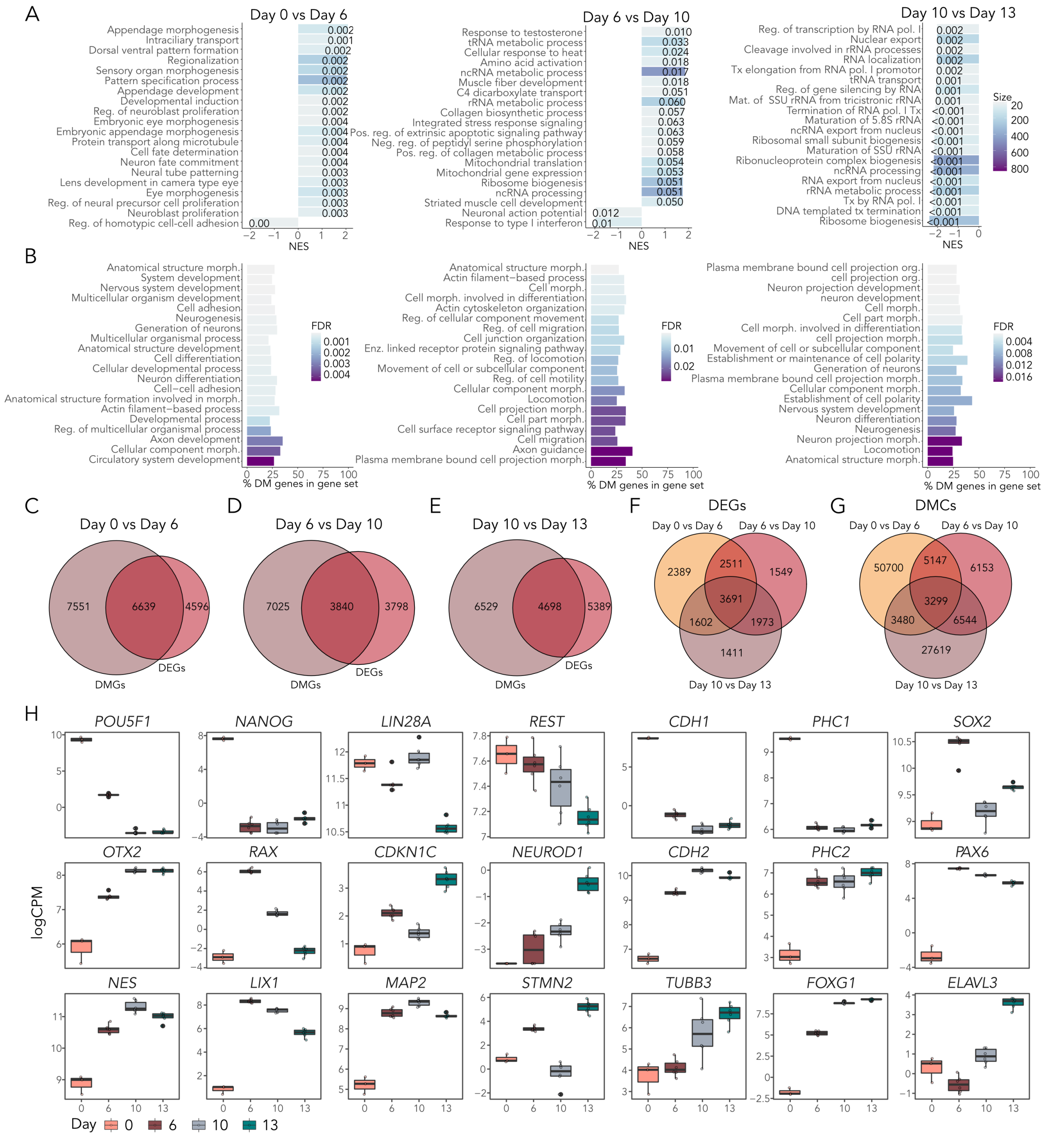

Figure S3. Gene expression profiles in control cells identify gene signatures consistent with neuronal differentiation. A) GSEA analysis of BPs based on a ranked list of gene expression changes between Day 0 and 6, Day 6 and 10 and Day 10 and 13. B) GOMETH analysis of top 20 BPs based on top 10 000 DMCs between Day 0 and 6, Day 6 and 10 and Day 10 and 13. C-E) Venn diagrams showing the overlap between differentially methylated genes (DMGs) and differentially expressed genes

(DEGs) between C) Day 0 and 6, D) Day 6 and 10 and E) Day 10 and 13. F) Venn diagram showing the overlap between DEGs between comparisons. G) Venn diagram showing the overlap between DMCs between comparisons. C-G) Genes or CpGs with adjusted p-value < 0.01 were considered significant. H) Gene expression (GE) levels (logCPM) of selected genes from loss of pluripotency towards neural induction and self-patterning.

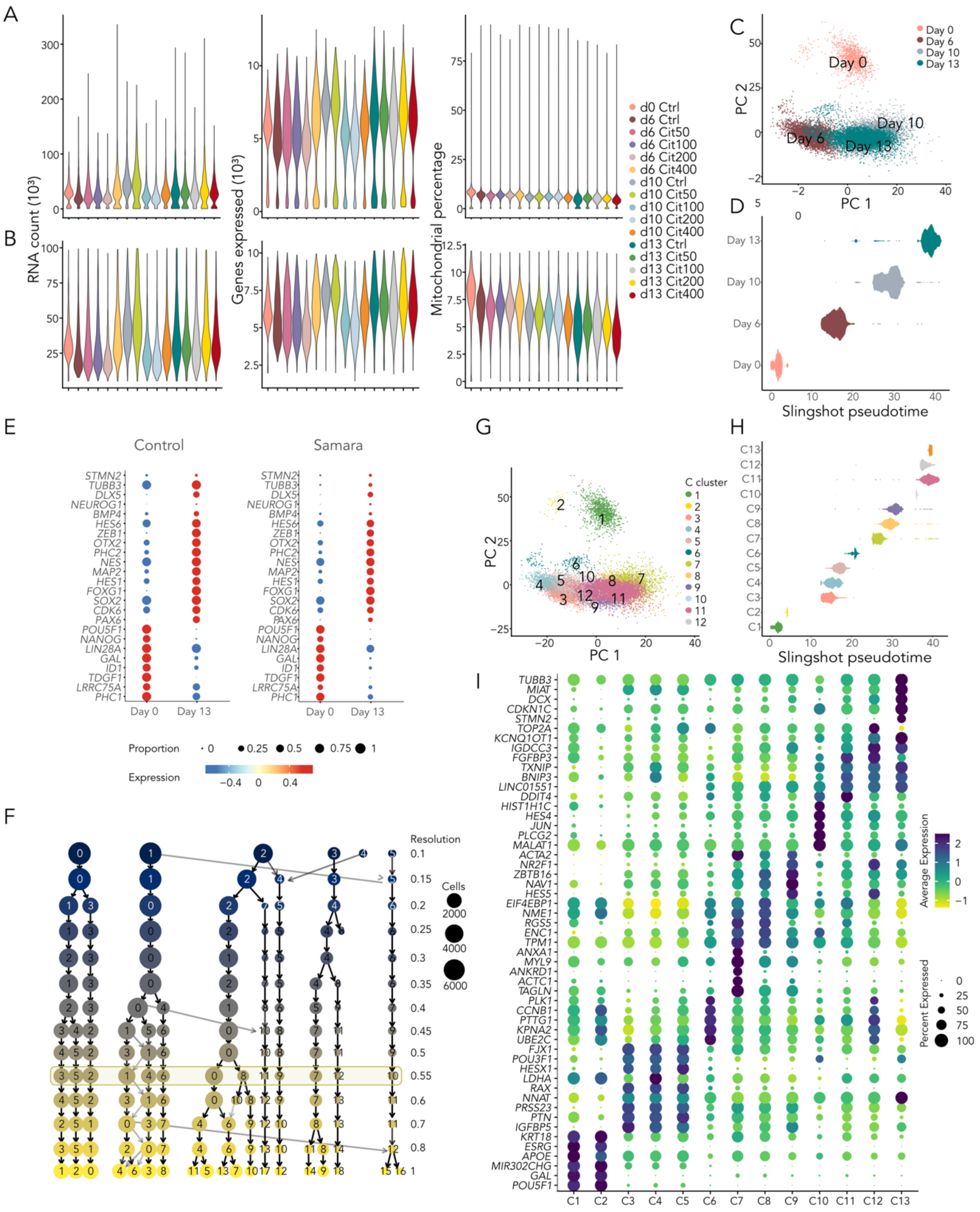

Figure S4. scRNA-seq data quality control and clustering of cells, related to Figure 5. A-B) Violin plots showing the RNA count, number of expressed genes and percentage mitochondrial genes present per cell A) before and B) after filtering out low quality cells. C) Principal component analysis (PCA)

displaying all cells colored by differentiation day. D) Cells were ordered according to Slingshot pseudotime and colored by differentiation day. E) Bubble plot showing the expression of selected markers at Day 0 and Day 13 in control cells and cells from Samara and Spildrejorde et al.(Samara et al., 2022). F) Visualization of the number of resulting Clustree-clusters at resolutions 0.1 to 1. Resolution 0.55 was chosen for downstream analysis, resulting in 13 citalopram (C) clusters. G) PCA colored by C clusters. H) Cells were ordered according to Slingshot pseudotime and colored by C clusters. I) Bubble plot showing the expression of the top 5 differentially expressed genes per C cluster.

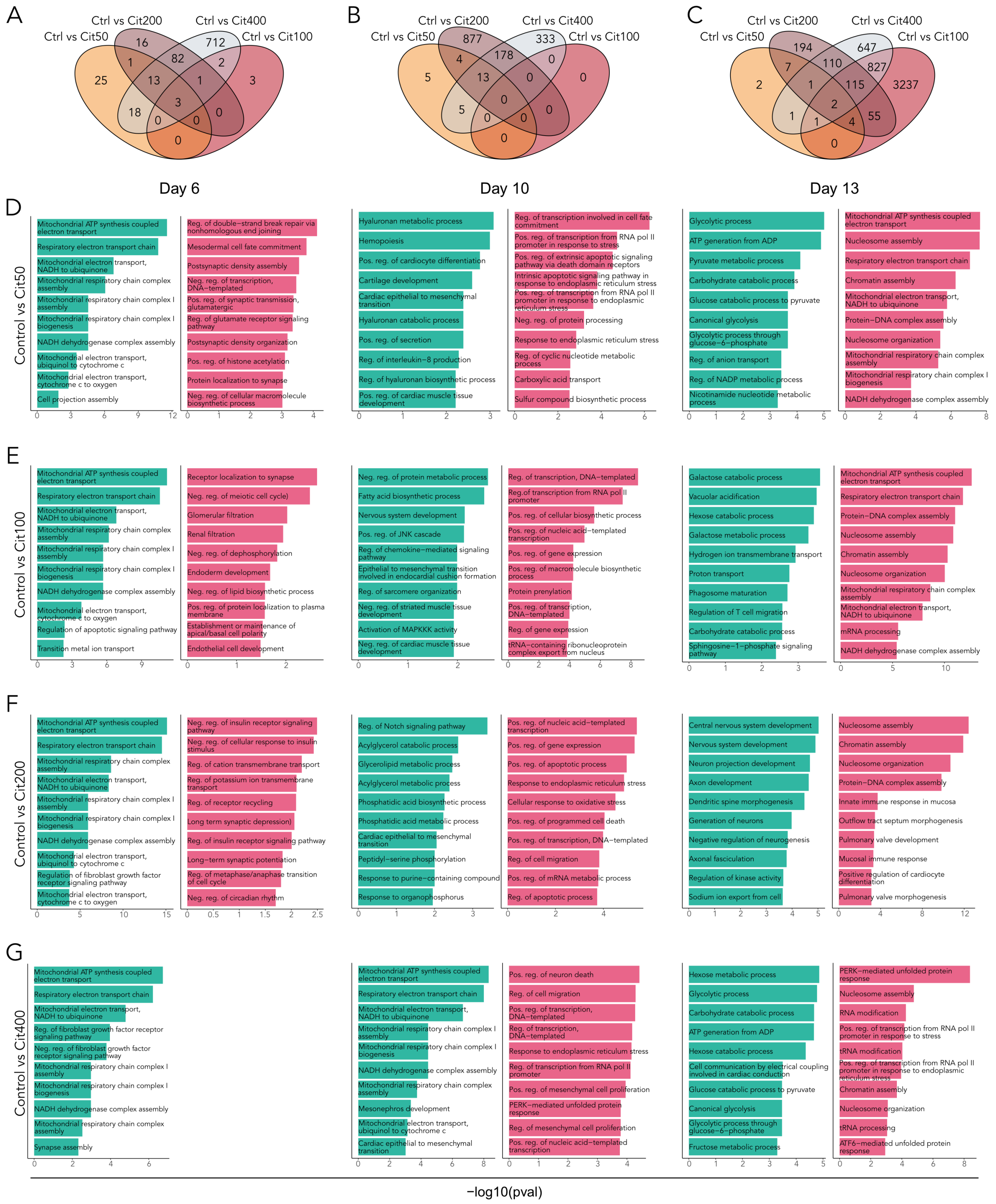

Figure S5. Pairwise citalopram-control comparisons, related to Figure 6A. A-C) Venn diagrams showing the overlap between differentially expressed genes derived from bulk RNA-seq at A) Day 6, B) Day 10 and C) Day 13. D-G) Top 10 upregulated (green) and downregulated (pink) BPs among

single cell DEGs at Day 6 (left), Day 10 (middle) and Day 13 (right) between D) Cit50, E) Cit100, F) Cit200 or G) Cit400 cells compared to control cells.

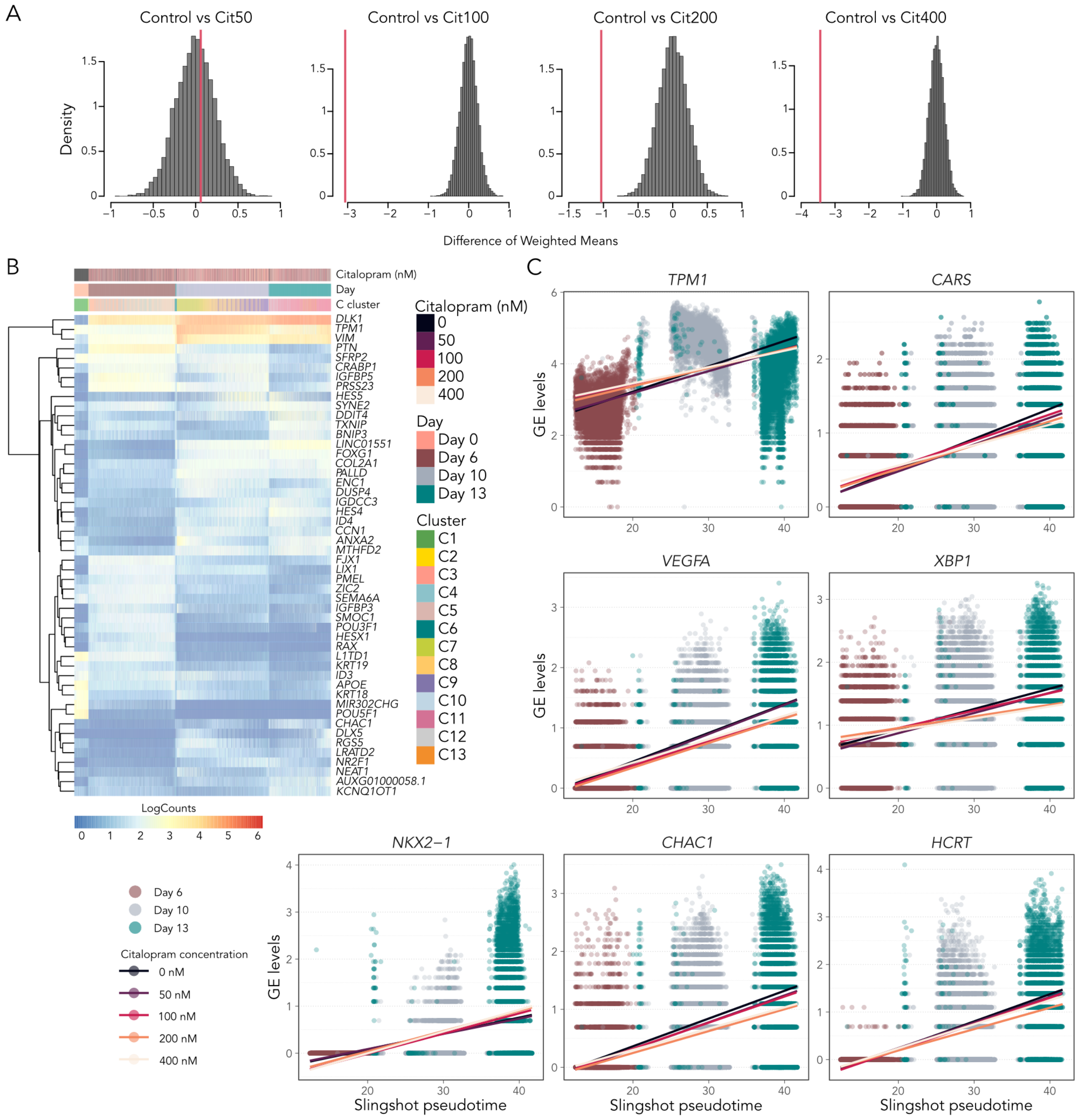

Figure S6. Slingshot pseudotime analysis, related to Figure 6B-C. A) Permutation test showing the differences in the weighted means of the between Slingshot pseudotime values of control cells and cells exposed to 50, 100, 200 or 400 nM citalopram. B) Top 50 temporally expressed genes in cells (columns) ordered by Slingshot pseudotime. C) GE levels (logcounts) of genes *TPM1*, *CARS*, *VEGFA*, *XBP1*, *NKX2-1*, *CHAC1* and *HRCT*, selected from the top 100 temporally expressed genes which responded differently in citalopram-exposed cells compared to control cells across Slingshot pseudotime at Day 6-13. One point represents one cell, and the lines represent the average GE for each citalopram concentration across pseudotime.

References

Amezquita, R. A., Lun, A. T. L., Becht, E., Carey, V. J., Carpp, L. N., Geistlinger, L., et al. (2020). Orchestrating single-cell analysis with Bioconductor. *Nature Methods* 17, 137–145. doi: 10.1038/s41592-019-0654-x

Aran, D., Looney, A. P., Liu, L., Wu, E., Fong, V., Hsu, A., et al. (2019). Reference-based analysis of lung single-cell sequencing reveals a transitional profibrotic macrophage. *Nature Immunology 2019 20:2* 20, 163–172. doi: 10.1038/s41590-018-0276-y

Aryee, M. J., Jaffe, A. E., Corrada-Bravo, H., Ladd-Acosta, C., Feinberg, A. P., Hansen, K. D., et al. (2014). Minfi: A flexible and comprehensive Bioconductor package for the analysis of Infinium DNA methylation microarrays. *Bioinformatics* 30, 1363–1369. doi: 10.1093/bioinformatics/btu049

Bushnell, B. (2014). *BBMap: A Fast, Accurate, Splice-Aware Aligner*. Available at: https://www.osti.gov/biblio/1241166

Chang, W., J, C., J, A., C, S., B, S., Y, X., et al. (2023). *shiny: Web Application Framework for R*. Available at: https://CRAN.R-project.org/package=shiny

Garnier, S., Ross, N., Rudis, R., Camargo, A. P., Sciaini, M., and Scherer, C. (2023). *viridis(Lite) - Colorblind-Friendly Color Maps for R. viridis package version 0.6.4.* Available at: https://sjmgarnier.github.io/viridis/

Hansen, K. D. (2016). *IlluminaHumanMethylationEPICmanifest: Manifest for Illumina's EPIC methylation arrays*. Available at: https://bitbucket.com/kasperdanielhansen/Illumina_EPIC

Hao, Y., Stuart, T., Kowalski, M. H., Choudhary, S., Hoffman, P., Hartman, A., et al. (2024). Dictionary learning for integrative, multimodal and scalable single-cell analysis. *Nat. Biotechnol.* 42, 293–304. doi: 10.1038/s41587-023-01767-y

Kassambara, A. (2020). *ggpubr: "ggplot2" Based Publication Ready Plots*.

Kim, D., Langmead, B., and Salzberg, S. L. (2015). HISAT: A fast spliced aligner with low memory requirements. *Nature Methods* 12, 357–360. doi: 10.1038/nmeth.3317

Kolde, R. (2019). *pheatmap: Pretty Heatmaps*.

Liao, Y., Smyth, G. K., and Shi, W. (2014). FeatureCounts: An efficient general purpose program for assigning sequence reads to genomic features. *Bioinformatics* 30, 923–930. doi: 10.1093/bioinformatics/btt656

McCarthy, D. J., Campbell, K. R., Lun, A. T. L., and Wills, Q. F. (2017). Scater: pre-processing, quality control, normalization and visualization of single-cell RNA-seq data in R. *Bioinformatics* 33, 1179. doi: 10.1093/bioinformatics/btw777

Ouyang, J. F., Kamaraj, U. S., Cao, E. Y., and Rackham, O. J. L. (2021). ShinyCell: simple and sharable visualization of single-cell gene expression data. *Bioinformatics* 37, 3374–3376. doi: 10.1093/bioinformatics/btab209

Phipson, B., Maksimovic, J., and Oshlack, A. (2016). MissMethyl: An R package for analyzing data from Illumina's HumanMethylation450 platform. *Bioinformatics* 32, 286–288. doi: 10.1093/bioinformatics/btv560

Rainer, J. (2017). *EnsDb.Hsapiens.v86: Ensembl based annotation package*.

Ritchie, M. E., Phipson, B., Wu, D., Hu, Y., Law, C. W., Shi, W., et al. (2015). Limma powers differential expression analyses for RNA-sequencing and microarray studies. *Nucleic Acids Research* 43, e47. doi: 10.1093/nar/gkv007

Robinson, M. D., McCarthy, D. J., and Smyth, G. K. (2010). edgeR: a Bioconductor package for differential expression analysis of digital gene expression data. *Bioinformatics* 26, 139–140. doi: 10.1093/bioinformatics/btp616

Samara, A., Spildrejorde, M., Sharma, A., Gervin, K., Lyle, R., and Eskeland, R. (2022). A multi-omics approach to visualize early neuronal differentiation from hESCs in 4D. *iScience* 25, 105279. doi: 10.1016/j.isci.2022.105279

Street, K., Risso, D., Fletcher, R. B., Das, D., Ngai, J., Yosef, N., et al. (2018). Slingshot: Cell lineage and pseudotime inference for single-cell transcriptomics. *BMC Genomics* 19, 1–16. doi: 10.1186/s12864-018-4772-0/figures/5

Stuart, T., Butler, A., Hoffman, P., Hafemeister, C., Papalexi, E., Mauck, W. M., et al. (2019). Comprehensive Integration of Single-Cell Data. *Cell* 177, 1888-1902.e21. doi: 10.1016/j.cell.2019.05.031/attachment/2f8b9ebe-54e6-43eb-9ef2-949b6bda8ba2/mmc3.pdf

Subramanian, A., Tamayo, P., Mootha, V. K., Mukherjee, S., Ebert, B. L., Gillette, M. A., et al. (2005). Gene set enrichment analysis: A knowledge-based approach for interpreting genome-wide expression profiles. *Proceedings of the National Academy of Sciences* 102, 15545–15550. doi: 10.1073/pnas.0506580102

Team, T. B. D. (2023). *BSgenome.Hsapiens.UCSC.hg38: Full genomic sequences for Homo sapiens (UCSC genome hg38)*.

Wickham, H. (2016). *ggplot2: Elegant Graphics for Data Analysis*. Springer-Verlag New York. Available at: https://ggplot2.tidyverse.org

Wickham, H., Averick, M., Bryan, J., Chang, W., McGowan, L., François, R., et al. (2019). Welcome to the Tidyverse. *Journal of Open Source Software* 4, 1686. doi: 10.21105/joss.01686

Zappia, L., and Oshlack, A. (2018). Clustering trees: a visualization for evaluating clusterings at multiple resolutions. *GigaScience* 7. doi: 10.1093/gigascience/giy083
